# Metaphenomic Responses of a Native Prairie Soil Microbiome to Moisture Perturbations

**DOI:** 10.1128/mSystems.00061-19

**Published:** 2019-06-11

**Authors:** Taniya Roy Chowdhury, Joon-Yong Lee, Eric M. Bottos, Colin J. Brislawn, Richard Allen White, Lisa M. Bramer, Joseph Brown, Jeremy D. Zucker, Young-Mo Kim, Ari Jumpponen, Charles W. Rice, Sarah J. Fansler, Thomas O. Metz, Lee Ann McCue, Stephen J. Callister, Hyun-Seob Song, Janet K. Jansson

**Affiliations:** a Earth and Biological Sciences Directorate, Pacific Northwest National Laboratory, Richland, Washington, USA; b National Security Directorate, Pacific Northwest National Laboratory, Richland, Washington, USA; c Division of Biology, Kansas State University, Manhattan, Kansas, USA; University of British Columbia

**Keywords:** metaphenome, metatranscriptome, multi-omics, soil microbiome

## Abstract

Climate change is causing shifts in precipitation patterns in the central grasslands of the United States, with largely unknown consequences on the collective physiological responses of the soil microbial community, i.e., the metaphenome. Here, we used an untargeted omics approach to determine the soil microbial community’s metaphenomic response to soil moisture and to define specific metabolic signatures of the response. Specifically, we aimed to develop the technical approaches and metabolic mapping framework necessary for future systematic ecological studies. We collected soil from three locations at the Konza Long-Term Ecological Research (LTER) field station in Kansas, and the soils were incubated for 15 days under dry or wet conditions and compared to field-moist controls. The microbiome response to wetting or drying was determined by 16S rRNA amplicon sequencing, metatranscriptomics, and metabolomics, and the resulting shifts in taxa, gene expression, and metabolites were assessed. Soil drying resulted in significant shifts in both the composition and function of the soil microbiome. In contrast, there were few changes following wetting. The combined metabolic and metatranscriptomic data were used to generate reaction networks to determine the metaphenomic response to soil moisture transitions. Site location was a strong determinant of the response of the soil microbiome to moisture perturbations. However, some specific metabolic pathways changed consistently across sites, including an increase in pathways and metabolites for production of sugars and other osmolytes as a response to drying. Using this approach, we demonstrate that despite the high complexity of the soil habitat, it is possible to generate insight into the effect of environmental change on the soil microbiome and its physiology and functions, thus laying the groundwork for future, targeted studies.

**IMPORTANCE** Climate change is predicted to result in increased drought extent and intensity in the highly productive, former tallgrass prairie region of the continental United States. These soils store large reserves of carbon. The decrease in soil moisture due to drought has largely unknown consequences on soil carbon cycling and other key biogeochemical cycles carried out by soil microbiomes. In this study, we found that soil drying had a significant impact on the structure and function of soil microbial communities, including shifts in expression of specific metabolic pathways, such as those leading toward production of osmoprotectant compounds. This study demonstrates the application of an untargeted multi-omics approach to decipher details of the soil microbial community’s metaphenotypic response to environmental perturbations and should be applicable to studies of other complex microbial systems as well.

## Abstract (precis)

Climate change is predicted to result in increased drought extent and intensity in the highly productive, former tallgrass prairie region of the continental United States. These soils store large reserves of carbon. The decrease in soil moisture due to drought has largely unknown consequences on soil carbon cycling and other key biogeochemical cycles carried out by soil microbiomes. In this study, we found that soil drying had a significant impact on the structure and function of soil microbial communities, including shifts in expression of specific metabolic pathways, such as those leading toward production of osmoprotectant compounds. This study demonstrates the application of an untargeted multi-omics approach to decipher details of the soil microbial community’s metaphenotypic response to environmental perturbations and should be applicable to studies of other complex microbial systems as well.

## INTRODUCTION

Climate change is predicted to result in increasing drought frequency and more infrequent, but higher-intensity, rainfall events in the highly fertile and agriculturally productive grasslands of the central United States (1). The southwestern and south-central grassland regions of North America are predicted to experience reduced precipitation in contrast to predicted higher precipitation in the central and northern grasslands (2, 3). As precipitation regimes with fewer, but larger, events amplify fluctuations in soil water content, the consequences in mesic grassland ecosystems will be prolonged dry periods between rainfall events and an increase in the length and occurrence of drought stress (4). These grassland soils store large amounts of carbon, up to 39% of the organic carbon stocks in the conterminous United States (5), and the fate of this stored C is uncertain as climate changes. Reportedly, grasslands in the U.S. Great Plains function as net carbon sinks (6, 7) but can become net carbon sources under drought (7, 8). Results from a series of modeling experiments simulating various drought magnitudes showed that short-term droughts caused greater carbon loss from grassland ecosystems (9, 10). However, contrasting results were obtained from a long-term rainfall intensity manipulation experiment at the Konza Long-Term Ecological Research (LTER) field station in Kansas (11), where soil microbial biomass and carbon use efficiency were higher in the treatments that had an extended duration between rainfall events, compared to ambient rainfall frequency. This study suggested that shifts in the soil microbiome following drying could result in a reduction in soil carbon loss and hypothesized that this could be due to an ecological selection for microbial species that are adapted to low-soil-water conditions (11).

It is well known that bacteria have evolved various life strategies to cope with soil desiccation, such as osmoregulation, dormancy/reactivation, and extracellular enzyme synthesis to accumulate substrates (12). As soil matric potentials become more negative, osmotic adjustments allow microbial cells to maintain turgor and function (13). Therefore, we predicted that soil drying would result in shifts in metabolic pathways and metabolites for osmoregulation (13). Understanding the physiological responses of the soil microbiome to changes in soil moisture is ultimately important for predicting and managing impacts of climate change on soil biogeochemistry (14) and grassland ecosystem productivity (15–17).

To date, most studies of soil microbial responses to wetting and drying have largely relied on bulk measurements of soil respiration, without knowledge of the underlying details of metabolic pathways that result in the measured release of CO_2_. More recently, 16S rRNA gene sequencing (16S) and metagenomic (MG) sequencing have revealed the identities and potential functions of members of soil microbial communities. However, bulk DNA extraction methods can include DNA from dead cells and dormant cells that are not actively growing at the time of extraction. Importantly, dormant cells represent a seed bank that can be resuscitated to an active state if the environmental conditions are suitable. Thus, a critical knowledge gap exists about how indigenous, active, and naturally interacting members of soil microbial communities respond to changes in soil moisture by changing levels of gene expression, as measured by metatranscriptomics (MT).

Here, we aimed to determine the metabolic pathways underpinning the soil microbial community’s phenotypic response to wetting and drying, i.e., to determine the metaphenome (18), using native prairie soils collected from three locations at the Konza LTER field station in Kansas (see Fig. S1 in the supplemental material). The moisture perturbations used in this study are particularly relevant as this central mesic grassland region is at the crossroads of predicted precipitation regime changes (2, 19). Our central hypothesis was that wetting and drying would result in consistent shifts in specific metabolic pathways utilized by the soil microbiome across different prairie soil field locations. Our simple experimental design, with three field locations and three treatments (control, wet, and dry), enabled us to optimize the omics and modeling approaches necessary to determine key metabolic pathways expressed by active members of the prairie soil microbiome and to determine how those pathways were impacted by changes in soil moisture. For example, we predicted that there would be similar shifts across field locations in expression of metabolic pathways and production of metabolites for adaptation to soil drying, such as an increase in pathways for osmolyte production (13), as also shown in studies using biocrust isolates (20). By further integration across omics data types using reaction network approaches, we aimed to use this information to help define the metaphenome.

10.1128/mSystems.00061-19.1 FIG S1 Topographic map of the Konza Native Prairie Long-Term Ecological Research station showing locations of the three soil sampling sites (A, B, and C) in this study. Download FIG S1, JPG file, 0.6 MB. Copyright © 2019 Roy Chowdhury et al. 2019 Roy Chowdhury et al. This content is distributed under the terms of the Creative Commons Attribution 4.0 International license.

## RESULTS

Native prairie soils were collected in triplicate from three field locations (soils A, B, and C; see Fig. S1 in the supplemental material) across a landscape gradient at the Konza Long-Term Ecological Research (LTER) site in Kansas (11). The soil samples were subjected to contrasting moisture perturbations, i.e., wetting to saturation or desiccation by air drying, over a 15-day period. Soils from the three locations differed in their initial gravimetric water contents and soil texture (Table S1). This allowed us to test the influence of changes in soil moisture content on the soil metaphenome using soils with different initial water contents, i.e., at time zero (Fig. S2a). Soil from site B had the lowest water content (18%) and the highest clay content (74%). Soils A and C had similar water contents (37% and 31%, respectively), although soil C had higher clay content than soil A. Concentrations of nitrate (<0.06 ppm) and sulfate (2.77 to 5.45 ppm), two of the major anions measured, were low in the three soils. Total carbon-to-nitrogen ratios were comparable between the three soils and ranged from 12 to 18.

10.1128/mSystems.00061-19.2 FIG S2 Soil moisture content and respiration measurements during soil incubations. (a) Changes in water content during the incubation period. (b) Changes in cumulative soil respiration (μmol CO_2_ g^−1^ [dry weight] soil) in response to shifts in moisture conditions over a 15-day incubation in soils A, B, and C. Data shown are means ± standard errors (*n* = 3). Pairwise comparisons in soil B (dry versus wet, *P* = 0.028, and dry versus control, *P* = 0.017). Pairwise comparisons in soil C (dry versus wet, *P* = 0.041, and dry versus control, *P* = 0.045). Download FIG S2, JPG file, 0.3 MB. Copyright © 2019 Roy Chowdhury et al. 2019 Roy Chowdhury et al. This content is distributed under the terms of the Creative Commons Attribution 4.0 International license.

10.1128/mSystems.00061-19.6 TABLE S1 Physicochemical properties of soils collected from three locations (A, B, and C) at the Kansas Native Prairie LTER station. Download Table S1, DOCX file, 0.01 MB. Copyright © 2019 Roy Chowdhury et al. 2019 Roy Chowdhury et al. This content is distributed under the terms of the Creative Commons Attribution 4.0 International license.

We measured soil respiration during the 15-day incubation (Fig. S2b). Carbon dioxide release (μmol CO_2_ g^−1^ [dry weight] soil) was significantly (*P* < 0.05) higher in response to drying than that in the wet and control treatments in soils B and C. The amounts of CO_2_ released at each time point were similar in the wet and control treatments. Although we did not measure soil oxygen levels, the increase in respiration in the dry soils could be indicative of higher rates of oxidation of organic substrates due to higher oxygen availability. Conversely, the lack of difference suggests that the moisture levels in the control soils were sufficiently high that they could not be distinguished from the wet treatments when additional water was added.

### Soil microbial community shifts with change in soil moisture.

Although we detected both *Bacteria* and *Archaea* in our samples, based on 16S sequencing, the majority of the sequences corresponded to *Bacteria*. We recognize that fungi are also important in C cycling, but we did not target fungi in these experiments. Therefore, the results presented below are focused on *Bacteria*.

**FIG 1:**
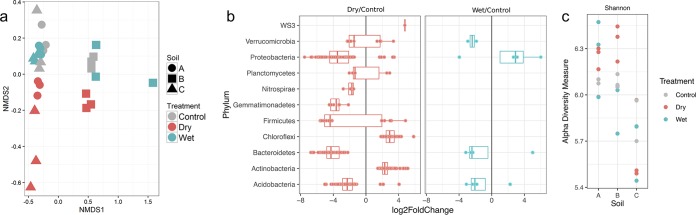
Soil microbiome response to wetting and drying. (a) Nonmetric multidimensional scaling (NMDS) plot of Bray-Curtis dissimilarities showing the microbial community structure estimated by 16S rRNA gene sequencing of soils collected from 3 Kansas prairie field locations. Site (A, circles; B, squares; C, triangles) and treatment (blue, wet; red, dry; gray, ambient field-moisture control). The stress value for the NMDS is 0.076. All sequence count data were normalized to the upper 75th quantile. (b) Differential abundances of OTUs within phyla that were observed to significantly shift in response to drying and wetting relative to continuous moisture control conditions (log_2_ fold change, adjusted *P* value < 0.01). (c) Alpha diversity (Shannon’s index) of the soil microbiome in soils A, B, and C, for control (gray), dry (red), and wet (blue) treatments.

By multivariate statistical analysis of the 16S data, we found that soil drying had a significant impact on bacterial *β*-diversity, as shown on the second axis of the nonmetric multidimensional scaling (NMDS) plot, which clearly separates all of the dried samples from the rest (Fig. 1a). In contrast, the wet samples clustered together with the control soils in ordination space, indicating that excess soil moisture had negligible impact on microbial community composition. Samples from the same soil location also grouped together, indicating that soil-specific factors were likely driving community composition.

Across all soil locations, there were significant (*P* < 0.01) shifts in specific taxa in response to drying, with significant decreases in operational taxonomic units (OTUs) corresponding to *Acidobacteria*, *Bacteroidetes*, *Gemmatimonadetes*, *Nitrospirae*, and *Proteobacteria* (Fig. 1b). Taxa that significantly increased after drying included members of the *Actinobacteria*, *Chloroflexi*, *Proteobacteria*, and WS3 candidate phylum. There were fewer significant shifts in taxa following wetting. Taxa that decreased after wetting included members of the *Verrucomicrobia*, *Bacteroidetes*, and *Acidobacteria*, while members of the *Proteobacteria* significantly increased (Fig. 1b). Drying also resulted in significant shifts in α-diversity (Kruskal-Wallis, *P* > 0.05) (Fig. 1c).

At a finer level of taxonomic resolution, the different field locations exhibited site-specific shifts in several OTUs in response to wetting or drying. Figure S3 shows specific OTUs that significantly (*P* < 0.01) increased or decreased in response to wetting or drying for each of the three soil locations. For example, members of the *Xanthomonadaceae, Sphingomonadaceae,* and *Chitinophagaceae* families consistently decreased in abundance following drying across all 3 soil locations. These families are comprised of common decomposers. Soil C had few OTUs that increased after drying, whereas soils A and B had increases in members of commonly stress-tolerant *Actinobacteria*, such as *Micromonosporaceae, Gaiellaceae, Frankiaceae,* Dolo_23, and an unidentified family (Fig. S3a). Note that there were no significant shifts in taxa following wetting in soil C (Fig. S3b, data not shown), whereas soils A and B exhibited few and inconsistent shifts (Fig. S3b).

10.1128/mSystems.00061-19.3 FIG S3 Impact of wetting and drying on soil microbial diversity and abundance of taxa. (a) Alpha-diversity indices determined based on 16S rRNA sequencing of soils from the three soils A, B, and C in response to wet and dry perturbations. (b) Differential abundance of OTUs observed in response to wetting in soils A and B; none were observed for soil C. Download FIG S3, JPG file, 0.3 MB. Copyright © 2019 Roy Chowdhury et al. 2019 Roy Chowdhury et al. This content is distributed under the terms of the Creative Commons Attribution 4.0 International license.

### Transcriptional response to changes in soil moisture.

**FIG 2:**
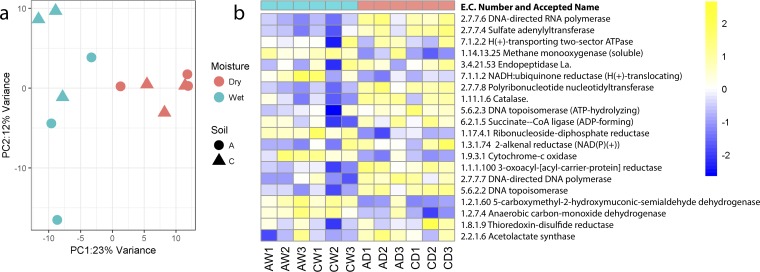
Response of soil metatranscriptome to moisture perturbations. (a) Metatranscriptome data shown as a PCoA ordination of Bray-Curtis dissimilarities of sequence data categorized by function, i.e., Enzyme Commission (EC) number. Site (A, circles; C, triangles) and treatment (blue, wet; red, dry). All sequence count data were normalized to the upper 75th quantile. (b) Heat map showing the top 20 most abundant transcripts (ECs) observed under dry relative to wet conditions. The *x* axis indicates soil sample (A or C), treatment (W, wet; D, dry), and replicate (1, 2, or 3). Moisture conditions are indicated by the header row in blue (wet) or red (dry). The color gradient for each cell is scaled to a log_2_ fold change of −2 to 2.

We obtained an average of 1,857 transcripts, annotated as Enzyme Commission (EC) numbers, from soils A and C. Unfortunately, we did not obtain sufficient RNA from site B to include that location in the analysis. Ordination of the data revealed that gene expression was significantly influenced by soil moisture changes in both soils A and C (Fig. 2a). The majority, >97%, of the transcripts corresponded to rRNA, but there were sufficient transcripts to also assess shifts in expression of the functional genes. A heat map of the top 20 most abundant transcripts (ECs) that were grouped according to treatment (Fig. 2b) showed that most of the abundant transcripts were higher under dry than wet conditions. These included several transcripts involved in nucleotide metabolism, including DNA-directed RNA polymerase (EC 2.7.7.6), sulfate adenylyltransferase (EC 2.7.7.4), polyribonucleotide nucleotransferase (EC 2.7.7.8), DNA-directed DNA polymerase (EC 2.7.7.7), and DNA topoisomerase (EC 5.99.1.3). Other abundant transcripts that were higher or lower under wet or dry conditions included several for central and peripheral carbon metabolism pathways. In addition to the most dominant transcripts, we screened for transcripts that significantly differed under dry compared to wet conditions (Table S2). These included several ECs corresponding to enzymes involved in carbon metabolism, and for biosynthesis of secondary metabolites and sugars, indicating that the community was responding by increasing pathways for production of osmolytes. For example, transcript levels for genes involved in trehalose production were significantly higher after drying (Table S2). This included transcripts for genes encoding trehalose phosphatase (EC 3.1.3.12) (adjusted *P* value [*P*_adj_] = 0.033 and 0.046 in soils A and C, respectively) and trehalose synthase (EC 5.4.99.16) (*P*_adj_ = 0.014 and 0.000 in soils A and C, respectively). Additional transcripts that significantly (*P*_adj_ < 0.05; see Table S2 for specific *P* values) increased after drying were the following: for soil A, pyruvate carboxylase (EC 6.4.1.1) and a hydrolase (EC 3.4.24.25), and for soil C, 5-carboxymethyl-2-hydroxymuconic-semialdehyde dehydrogenase (EC 1.2.1.60) and malate dehydrogenase (EC 1.1.1.40). However, other transcripts involved in carbon metabolism were significantly lower (*P*_adj_ < 0.05) in both soils after drying, including pyruvate dehydrogenase (EC 1.2.2.2), a hydrolase (EC 3.1.4.4), and kojibiose phosphorylase (EC 2.4.1.230).

10.1128/mSystems.00061-19.7 TABLE S2 Transcripts showing significant changes in response to drying relative to the wet conditions in soils A and C. Results are outputs from differential analysis of metatranscriptome count data annotated as Enzyme Commission (EC) numbers. Columns show ECs, the average of the normalized count values divided by size factors taken over all samples (baseMean), the effect size estimate (log_2_ fold change), standard error estimate for the log_2_ fold change estimates (lfcSE), *P* values (pvalue), and adjusted *P* values (padj). All values with an FC of <1.5 and *P*_adj_ of <0.05 are considered significantly lower. All values with an FC of >1.5 and *P*_adj_ of <0.05 are considered significantly higher. Data were analyzed using the R package DESeq2. Download Table S2, DOCX file, 0.02 MB. Copyright © 2019 Roy Chowdhury et al. 2019 Roy Chowdhury et al. This content is distributed under the terms of the Creative Commons Attribution 4.0 International license.

**FIG 3:**
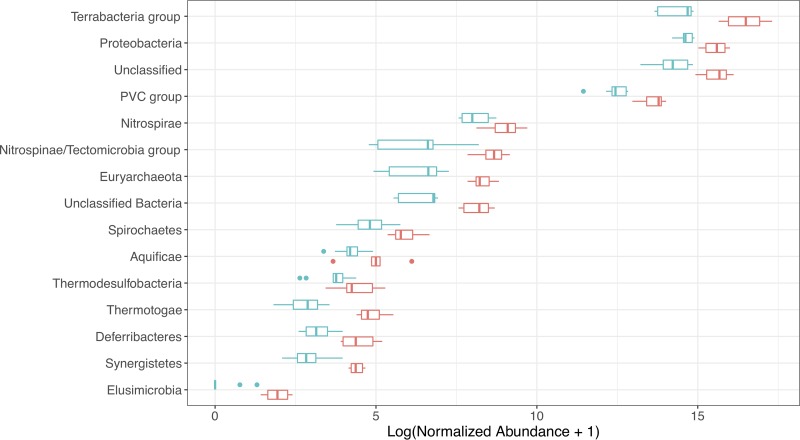
Transcriptional response of different phyla to soil wetting and drying. Log-normalized abundance of active members of the soil microbial community that significantly shifted in abundance in response to drying relative to the wet conditions, as revealed by mapping transcripts to contig-level taxonomies derived from the soil metagenome. All sequence count data were normalized to the upper 75th quantile. Log_10_ values of the normalized read counts are presented on the *x* axis, and taxa that shifted significantly in abundance at the phylum level are shown on the *y* axis. Changes in read count were considered significant for log_2_ fold change of >2.0 and *P*_adj_ of <0.05.

To determine which members of the community were active under the different soil moisture conditions, all of the metatranscriptomic reads from both soils A and C were mapped to contigs having phylogenetic assignments, and the read abundances were determined for each condition (Fig. 3). Most taxa had higher levels of expression under dry than wet conditions. Representatives of the *Terrabacteria* group, in particular, showed the highest levels of expression under both wet and dry conditions, although significantly higher under dry conditions (*P*_adj_ < 0.001). This large group encompasses nearly two-thirds of bacterial species, including *Actinobacteria*, *Firmicutes*, *Cyanobacteria*, *Chloroflexi*, and *Deinococcus*-*Thermus*.

### Shifts in metabolite profiles following changes in soil moisture.

**FIG 4:**
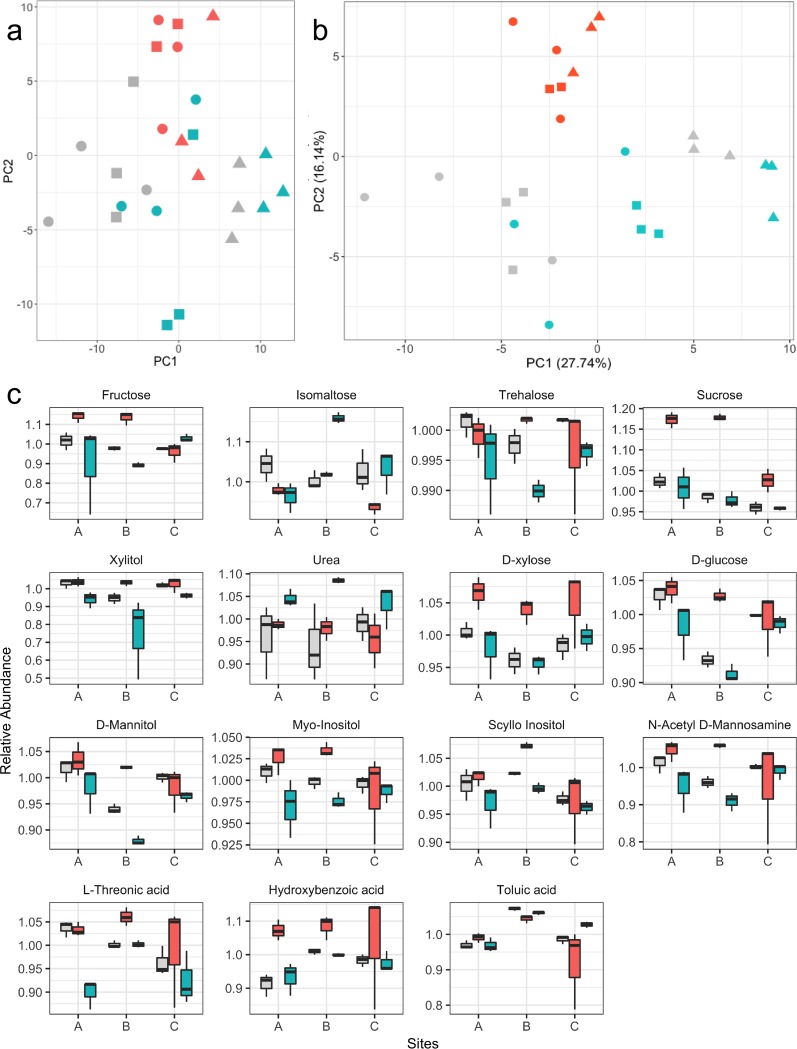
Impact of soil moisture treatments on the soil metabolome. (a) Global metabolome data shown as a projection pursuit principal-component analysis (PPCA) of all detected metabolites. Relative abundance data for metabolites were log_2_ transformed and median centered. The % on the axes represents the variance explained by each of the coordinates. Site (A, circles; B, triangles; C, squares) and treatment (gray, control; blue, wet; red, dry). (b) Metabolite data shown as a projection pursuit principal-component analysis (PPCA) of metabolites with significant treatment effects for at least one site. (c) Fifteen of 70 detected metabolites that changed significantly under the wet or dry treatments compared to control (*P* < 0.05, one-way ANOVA). Whiskers indicate the most extreme values within 1.5 multiplied by the interquartile region. Box, 25% quartile; median, 75% quartile. Pairwise comparisons of means to test treatment effects were performed after outlier removal.

Subsequently, we applied an untargeted metabolomics approach to determine the impact of soil wetting and drying on the soil metabolome. A total of 165 metabolites were detected, although only 70 could be identified in reference databases (see Table S3 for the full list of metabolites and *P* values). Global metabolome data shown as a projection pursuit principal-component analysis (PPCA) of all detected metabolites revealed a separation of the dry soils from the wet and control soils (Fig. 4a). This distinction was more evident by using PPCA of metabolites with significant treatment effects for at least one site (Fig. 4b), where the dry samples clearly clustered separately from the other samples. Fifteen of the 70 detected metabolites changed significantly (*P* < 0.05) under the wet or dry treatments compared to control (Fig. 4c). Soil desiccation resulted in consistently significant increases in several sugars and sugar alcohols across all 3 sampling locations (Fig. S4). In general, the relative abundances of simple sugars and carboxy acids (e.g., glucose, sucrose, fructose, mannose, xylose, trehalose, hydroxybenzoic acid, threonic acid, and toluic acid) were significantly higher after drying compared to the control soil (Fig. S4a). Other metabolites that increased in relative abundance after drying in individual locations included acids, amines, and alcohols (Fig. S4b to d).

10.1128/mSystems.00061-19.4 FIG S4 Box plots showing relative abundances of simple carbohydrates (a), acids (b), alcohols (c), and amines (d), in response to the dry or wet treatments compared to control conditions in soils A, B, and C. Relative abundance measurements were obtained at the end of incubation of samples (*t* = 15 days). Data were normalized by median centering and log_2_ transformed. Whiskers indicate the most extreme values within 1.5 multiplied by the interquartile region. Box, 25% quartile; median, 75% quartile. Pairwise comparisons of means to test treatment effects were performed after outlier removal. Download FIG S4, DOCX file, 0.4 MB. Copyright © 2019 Roy Chowdhury et al. 2019 Roy Chowdhury et al. This content is distributed under the terms of the Creative Commons Attribution 4.0 International license.

10.1128/mSystems.00061-19.8 TABLE S3 List of metabolites detected in soils in response to wetting and drying using GC-MS. Data reported are relative abundances of identified metabolites that were log_2_ transformed and median center normalized. *P* values for Bonferroni tests are included. Download Table S3, DOCX file, 0.02 MB. Copyright © 2019 Roy Chowdhury et al. 2019 Roy Chowdhury et al. This content is distributed under the terms of the Creative Commons Attribution 4.0 International license.

### Identification of reaction networks responding to soil wetting and drying.

**FIG 5:**
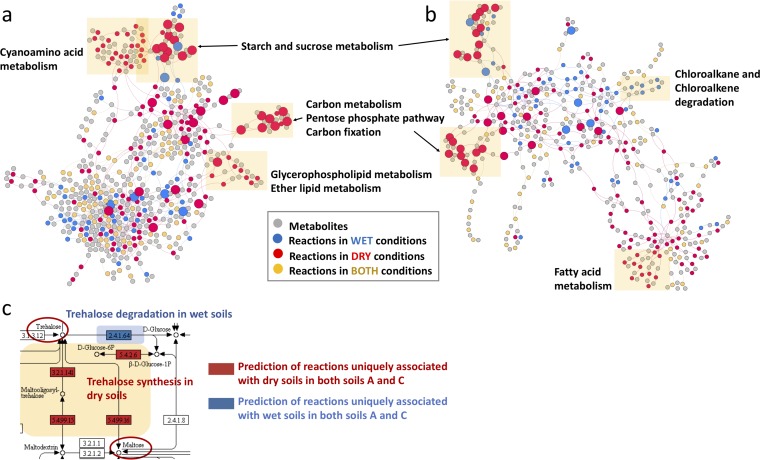
Prediction by MEMPIS of the moisture impact on biochemistry in native prairie soil. (a and b) Reaction-metabolite integrative bipartite networks for soils A (a) and C (b) are shown. Gray symbols indicate metabolites, and lines indicate relationships between reactions or metabolites based on KEGG annotation. Colored symbols indicate reactions that are more prevalent under specific incubation conditions: blue, wet; red, dry; yellow, both. Yellow shading highlights specific pathways that are more prevalent under dry conditions. Larger nodes represent the commonly predicted reactions in both soils A and C, while small nodes represent the uniquely predicted reactions in the individual soil. (c) Predicted reactions for trehalose synthesis and degradation in the starch and sucrose metabolism pathways are shown. Colored boxes indicate the predicted reactions uniquely associated with dry and wet conditions in both soils A and C.

To characterize the soil metaphenome, we incorporated metatranscriptomes and metabolites into a metabolic reaction network model (Fig. S5a). Using this method, we identified condition-specific reaction pathways that were activated under the dry and wet conditions, respectively, relative to the control conditions, in soils A and C (Fig. S5b). Based on a hierarchy of reference pathways described in the KEGG database, we classified the identified reactions into metabolite-reaction networks. Finally, we converted the identified metabolite-reaction networks into undirected bipartite graphs (Fig. 5). This graphical representation identified four clusters associated with dry conditions in soil A: (i) “starch and sucrose metabolism”; (ii) “cyanoamino acid metabolism”; (iii) “carbon metabolism, pentose phosphate pathway, and carbon fixation”; and (iv) “glycerophospholipid metabolism” and “ether lipid metabolism.” Metabolite-reaction networks in soil C under dry conditions also included “starch and sucrose metabolism” and “carbon metabolism, pentose phosphate pathway, and carbon fixation.” In addition, “fatty acid metabolism” and “chloroalkane and chloroalkene degradation” were uniquely found in soil C under dry conditions and wet conditions, respectively. In contrast, no such distinct clusters were observed in the wet samples from soil A. In both soils, we predicted reactions that were uniquely associated with wet or dry soils in both soils A and C (Fig. 5c). Interestingly, these reactions were for synthesis of trehalose in dry soils and trehalose degradation in wet soils.

10.1128/mSystems.00061-19.5 FIG S5 Metabolic networks and predicted reactions from metatranscriptome and metabolite data that were constructed using MEMPIS. (a) Workflow for the integration of metatranscriptomes and metabolite profile data into a metabolic network. (b) Bar plots showing the number of predicted and significantly changed reactions in a soil-moisture combination using the reference reaction modules in the Kyoto Encyclopedia of Genes and Genomes (KEGG) for soil A-dry, soil A-wet, soil C-dry, and soil C-wet conditions. Download FIG S5, JPG file, 0.2 MB. Copyright © 2019 Roy Chowdhury et al. 2019 Roy Chowdhury et al. This content is distributed under the terms of the Creative Commons Attribution 4.0 International license.

Thus, our approach to integrate the empirical observations from the soil metatranscriptome and metabolome into metabolic networks was in alignment with findings from the individual data sets and also highlighted specific reaction modules of potential significance under soil drought for further targeted investigations, e.g., cyanoamino acid metabolism observed only in soil A under dry conditions and chloroalkane and chloroalkene degradation observed only in soil C under wet conditions and trehalose metabolism in both soils.

The activated and enriched reaction modules for each soil-moisture treatment combination are presented individually, i.e., soil A-dry, soil A-wet, soil C-dry, and soil C-wet (Fig. S5b). Reaction module distributions between the two wet conditions in soils A and C showed a high correlation (i.e., correlation coefficient of 0.71), which implies that wet biochemistry was not very different across the two soil sites. However, there was a low correlation between reaction module distributions from the two soils after drying (i.e., correlation coefficient of 0.56), which implies that the effect of drying may be different depending on soil type or location. Conversely, the within-soil correlation between wet and dry treatments in soil A was 0.5 and in soil C was 0.62, also indicating a soil-specific response to such moisture shifts, such as cyanoanimo acid metabolism that was found only in soil A under dry conditions as mentioned above. These findings are counter to our overarching hypothesis that there would be similar metaphenomic responses across field locations to changes in soil moisture, other than those found for trehalose metabolism (Fig. 5c).

## DISCUSSION

We developed an untargeted omics approach to determine the soil microbial community’s metaphenomic response to soil moisture and to define specific metabolic signatures of the response. By incorporating both metatranscriptomics and metabolomics into the same metabolic reaction networks, we aimed to identify reaction pathways distinctively activated in response to the moisture perturbations and in an ecosystem-specific manner. We refer to these reaction pathways as being “context-specific” reaction modules, i.e., soil- and treatment-specific groups of reactions that are functionally related. Using this approach, we demonstrate that despite the high complexity of the soil habitat, it is possible to generate an integrated understanding of the effect of environmental change on the soil microbiome and the soil metaphenome.

Our field sampling across the Konza native prairie site took advantage of natural variability across the site, including locations that were naturally wetter or drier (see Fig. S1 and Table S1 in the supplemental material). Although the soil microbial community composition profiles, gene expression patterns, and metabolites shifted upon drying across all soil locations, we observed minor differences between wet and control soils. These results suggest that the soil microorganisms at our sampling sites were more adapted to wet conditions at the times of sampling. Our data also suggested that soil location was the primary driver and that change in soil moisture was a secondary driver of soil microbial composition and function. In contrast, in a recent study, we found that samples collected from the Konza prairie site at a maximum distance of 10 m from each other clustered together and separately from adjacent cultivated soils (21). In the same study, we also found that prairie soils from different states (Iowa and Wisconsin) exhibited similar soil microbiome structures compared to adjacent cultivated soils. Therefore, we proposed the concept of a prairie microbiome that was relatively consistent across locations (21). Previous studies that experimentally manipulated durations of drought and rainfall events in the Konza prairie offered a limited explanation of the variation in soil respiration due to moisture and implicated the whole-community microbial response as a major driver (22). Here, we find, however, the need to account for field-scale heterogeneity, in particular that of hydrology, across a site in order to better understand how the microbiome is impacted by changes in soil moisture.

Our results showed that soil drying, in particular, had significant impacts on all omics levels that we investigated, from community composition to gene expression and metabolite profiles. Although the Konza LTER site had not experienced a recent drought at the time of our sampling, legacy effects and microbial dormancy might explain some of our results. The central grassland regions experience drought every 20 to 30 years, and the last drought was recorded in the 2000s based on published data (23). Our short-term (15-day) drying experiment was designed to reveal the initial response of the soil microbiome to desiccation. However, longer-term experiments are needed to predict the metaphenomic response to periods of drought that can last months to years.

Increasing the soil moisture contents to saturation had little impact on the soil microbiome. Therefore, drying may have a more pronounced impact over a shorter time frame than wetting in the soils we studied. This could be due to negligible changes in abiotic conditions between the wet control and water-logged treatments. Alternatively, the lack of pronounced differences upon soil saturation relative to the control could be attributed to a soil microbiome acclimatized to higher antecedent soil moisture status due to frequent seasonal precipitation events. Indeed, soils A and C were located in low-lying regions of the landscape (Fig. S1) with relatively wet (∼30%) *in situ* soil moisture observed at the time of collection. In contrast, soil B had a lower gravimetric water content and was harder to wet up, presumably due to the higher clay content that would slow water permeation. Previous studies from sites adjacent to our field location found that when the soil microbiome was preadapted to heavy rainfall, there was a negligible response to laboratory saturation (14). This suggests that the historical soil moisture regime may impact current rates of microbial processes (e.g., respiration activity) by aggregating community-level traits that control carbon use efficiency, soil moisture sensitivity, and stress tolerance (24).

Our results corroborate other research that has reported relative decreases in the Gram-negative phyla *Proteobacteria*, *Verrucomicrobia*, and *Bacteroidetes* (12, 25–27) and increases in the Gram-positive phyla *Firmicutes* and *Actinobacteria* (25, 28–30) after soil drying. These shifts in relative abundance at the phylum level are driven by increases or decreases in abundances of specific groups within each respective phylum (12, 30), depending on their respective metabolic capacity to respond to the change in environmental conditions (27, 31). However, most of these phylum-level distinctions in relative abundance were not mirrored at the transcriptional level. Instead, drying increased the transcriptional activity across different phyla. We found that members of the *Terrabacteria* group and *Proteobacteria* were the most transcriptionally active in general and in response to soil drying. The *Terrabacteria* group consists of the Gram-positive *Actinobacteria* and *Firmicutes*, in addition to *Chloroflexi* and *Cyanobacteria* (32), several species of which are known to be desiccation and heat tolerant and have been reported under warmer (33) and drier (34) soil conditions. Additionally, several genera within the *Actinobacteria* are known to be capable of survival in arid soils (27, 30, 35), which supports our findings.

We performed an integrative analysis of the multi-omics data to generate metabolic networks activated in response to the soil moisture perturbations. The metabolite data were the most discriminating data set with the highest number of significant features that were consistently significantly different across the three sampling locations. In contrast, the metatranscriptome data were highly variable between sampling sites. One of the limitations of this study was that we obtained high-quality RNA from only 2 of the 3 field locations. Therefore, the significance of our metatranscriptome data was often difficult to assess, particularly since there were many opposite trends in the community expression data when comparing the two sites. This could be due to the transient nature of the transcripts that are highly dependent on *in situ* environmental variables that govern gene expression, as well as the relatively low depth of sequence coverage of the transcripts. The metabolite data were also limited because of the high number of unknown metabolites in the samples.

When we used our data-integrated metabolic network modeling approach, it was, however, possible to reveal specific pathways that were expressed in the soil microbial community as a response to moisture perturbation. In contrast to discrete data-reaction mapping, this framework extended the series of localized snapshots of information to a global metabolic reaction network scale, enabling the identification of context-specific reaction modules. Indeed, distributions of reaction modules among dry/wet soil A and dry/wet soil C were shown to be fundamentally different (Fig. S5). Importantly, beyond simple quantification of the cross-condition differences in biochemistry, our metabolic network analysis pointed out specific reactions that shifted in response to change in soil moisture and provides the basis for formulating new hypotheses to test for validation of model predictions. For example, we found that transcripts for cyanoamino acid metabolism were more prevalent after drying in soil A. Although the significance of cyanoamino acid accumulation is not known for soil microbes, transcription of cyanoamino acids has been shown to increase during salt stress in desert poplar trees (64), suggesting that these atypical amino acids are protective under desiccation conditions. This is a hypothesis to test in future experiments.

In summary, we were able to extract information about the specific physiological responses of the soil microbiome and metaphenome to moisture perturbations. We were able to capture a contingent of metabolic mechanisms that the soil bacterial community uses in response to water deficit such as alteration of metabolism, osmolyte biosynthesis, and transcription control. The integration of multiple omics technologies with metabolic pathway modeling now provides a richer knowledge base for better understanding of the soil metaphenome.

## MATERIALS AND METHODS

### Site description and sample collection.

Soil samples were collected at the Konza Prairie Biological Station (KPBS), a Long-Term Ecological Research (LTER) site representative of native tallgrass prairie in the Flint Hills of eastern Kansas, USA, from three field locations: site A, 39°06′11″N, 96°36′48″W, 339 meters above sea level (MAMS); site B, 39°04′39″N, 96°36′29″W, 413 MAMS; and site C, 39°04′20″N, 96°34′33″W, 415 MAMS (see Fig. S1 in the supplemental material). These sampling locations or sites remain undisturbed by agriculture and are dominated by perennial C4 grasses characteristic of native lowland tallgrass prairies in this region. Our selected sampling sites were burned annually (sites A and C) or at 4-year intervals (site B). The Flint Hills are generally characterized by shallow soils overlaying chert-bearing limestones and shales (36), and the site is classified as typical chernozem according to the Food and Agriculture Organizations (FAO) soils classification used by the United Nations and belongs to the Mollisol order of the U.S. Soil Taxonomy. The study soils have been previously reported as low-lying Irwin silty clay loams (fine, mixed mesic Pachic Argiustolls, USDA Natural Resources Conservation Service (NRCS) (11). The three soils used in this study represented locations across a natural hydrology gradient such that soils A and C were from the wetter areas and soil B was from a relatively drier area of the landscape (Fig. S1) and located adjacent to the Rainfall Manipulation Plots (RaMPs) at this LTER site (37, 38).

At each of the three sites, soil samples were obtained from three locations at least 10 m apart at 0- to 15-cm depth with a shovel. Roots and large rocks were removed; the soil was manually mixed, first in a 5-gal bucket and then in 1-gal Ziploc bags; and soil was composited into one bag for each site. Soils were immediately frozen under liquid nitrogen and shipped to the Pacific Northwest National Laboratory (PNNL), Richland, WA. Upon receipt at PNNL, the soils were quickly sieved through a 2-mm sieve and 50-g portions of the soils were aliquoted to 50-ml Falcon tubes and flash-frozen at −80°C to serve as baseline samples and replicates for the experiments described below.

### Experimental design and laboratory incubation.

We experimentally wetted and dried the soils collected from three field locations using a simple experimental design to determine the effects of extreme moisture shifts on the soil microbiome structure and function. Previously processed (as described above) soils were thawed and preincubated at 21°C for 48 h before the onset of treatments. After temperature equilibration, triplicate microcosms (20 g soil in 50-ml glass beakers) were subjected to three moisture conditions: wetted to saturation (addition of sterile deionized water), air dried to constant weight by evaporation, and maintained at ambient field-moist conditions. The 27 microcosms (3 sites × 3 treatments × 3 replicates) were maintained gravimetrically and incubated at 21°C in a biosafety cabinet (open to the atmosphere inside) for 15 days (Fig. S2b). For the wet treatment, water was added to the 3 soils such that the endpoint moisture contents for the saturation treatments allowed for excess (not inundated) unabsorbed water in the microcosms and were in the range of that reported for *in situ* delayed rainfall treatments at Konza Biological Station (14). We used percent water-filled pore spaces (% WFPS) to estimate water content and aeration status (39) using equations 1 and 2:

(1) $$\%\text{WFPS}=\frac{\text{gravimetric water content}\times \text{Db}}{\text{total soil porosity}}$$

(2) $$\text{soil porosity}=1-\left(\frac{\text{Db}}{\text{Dp}}\right)$$

where average soil bulk density (Db) = 1.1 g cm^−3^ and assumed particle density (Dp) = 2.65 g cm^−3^.

The final WFPS ranged from ∼56% to 84% in the saturation treatments and from ∼22% to 47% in the dry treatments compared to ∼33% to 68% in the control soils (Table S1). At the end of the experiment, subsamples from each replicate microcosm were collected for nucleic acid and metabolite extractions as described below. We refer to the saturated samples as “wet,” the air-dried samples as “dry,” and the field-moist soils as “control” where the weights were kept constant by addition of sterile water throughout the incubation period (Fig. S2b).

Soil respiration from the wet, dry, and control incubations was measured at regular intervals of 2 to 3 days using a G2301 Picarro GHG analyzer (Picarro, Sunnyvale, CA, USA) (Fig. S2b). Headspace gases of each sample were measured for 2 h, and fluxes were calculated from the change in concentration using the Ideal Gas Law and equation 3:

(3) $$F=(\frac{\text{dC}}{\text{dT}}\times \frac{V}{M}\times \frac{P}{\mathrm{RT}})$$

where *F* is the flux (μmol g soil^−1^ s^−1^), dC/dT is the rate of change in CO_2_ concentration (mol fraction s^−1^), *V* is the total volume (cm^3^) of microcosm and chamber, *M* is the dry weight of soil (g), *P* is the atmospheric pressure (kPa), *R* is the universal gas constant (8.3 × 10^3^ cm^3^ kPa mol^−1^ K^−1^), and *T* is the temperature of incubation (K).

### Nucleic acid extraction.

DNA was extracted from 0.25 g (wet weight) soil using the PowerSoil DNA isolation kit (Mo Bio Laboratories Inc., Carlsbad, CA) according to the manufacturer’s instructions, and extracts were quantified using the Qubit Fluorometer 2.0 (Invitrogen, Carlsbad, CA, USA) and checked for quality using the NanoDrop 2000 spectrophotometer (Thermo Scientific, Waltham, MA) and Bioanalyzer HS DNA chips (Agilent Technologies, Santa Clara, CA).

RNA was extracted from the soil samples (2 g) using the PowerSoil RNA isolation kit (Mo Bio Laboratories Inc., Carlsbad, CA) according to the manufacturer’s instructions. RNA was DNase treated with Turbo DNase (Life Technologies, Grand Island, NY, USA) at 37°C for 20 min. RNA was subsequently purified by phenol-chloroform extraction and recovered by precipitation with isopropanol. RNA was quantified using the Qubit Fluorometer 2.0 (Invitrogen, Carlsbad, CA, USA) and quality checked using a NanoDrop 2000 spectrophotometer (Thermo Scientific, Waltham, MA) and Bioanalyzer Pico RNA chips (Agilent Technologies, Santa Clara, CA). First-strand synthesis of cDNA from RNA was carried out using the SuperScript VILO cDNA synthesis kit (Life Technologies) according to the manufacturer’s protocol using a 1:1 blend of 1 μM random hexamers/decamers. Second-strand synthesis was performed using the NEBNext mRNA second-strand synthesis module and T4 gene 32 (New England Biolabs, Ipswich, MA) according to the manufacturer’s protocol at 16°C for 2 h. cDNA was purified using Agencourt AMPure XP beads (Beckman Coulter, Beverly, MA). The resulting cDNA was quantified and quality checked using the Agilent RNA 6000 Pico kit on a 2100 Bioanalyzer instrument (Agilent Technologies, Santa Clara, CA).

For Illumina library construction, DNA and cDNA were sheared to approximately 450 to 500 bp by using a Covaris M220 focused ultrasonicator instrument (Covaris M220 series; Woburn, MA). Sheared DNA fragments were end repaired, A tailed, and ligated to TruSeq adapters from Illumina according to the manufacturer’s instructions (Illumina, San Diego, CA). Adapter dimers were removed twice using Agencourt AMPure XP magnetic beads (Beckman Coulter, Danvers, MA). Libraries were checked for size and adapter dimers using a high-sensitivity DNA chip on a 2100 Bioanalyzer (Agilent Technologies, Santa Clara, CA) and quantified by qPCR on a StepOne real-time PCR system (Thermo Fisher Scientific) using the KAPA library quantification kit (KAPA Biosystems, Wilmington, MA) according to the manufacturer’s instructions. Fragments were sequenced using the Illumina MiSeq and HiSeq sequencing platforms (3 lanes) using the V3 chemistry (Illumina, San Diego, CA) for 300 cycles, generating 2 × 250-bp paired-end reads and ∼10 million reads per sample.

For the metatranscriptome data analysis from the moisture perturbation experiment, we focused on soils A and C, because of difficulties in obtaining sufficient high-quality RNA from site B. We were also able to obtain high-quality RNA and sequence the metatranscriptomes from only one of the 3 replicates from the soil A and C controls.

### 16S rRNA gene sequencing and analysis.

PCR amplification of the V4 region of the 16S rRNA gene was performed using the protocol developed by the Earth Microbiome Project (40) and as previously described (41), with the exception that the 12-base barcode sequence was included in the forward primer. Amplicons were sequenced on an Illumina MiSeq using the 500-cycle MiSeq reagent kit v2 (Illumina, San Diego, CA) according to the manufacturer’s instructions.

Raw sequence reads were demultiplexed with EA-Utils (42) with zero mismatches allowed in the barcode sequence. Reads were quality checked with BBDuk2 (43) to remove adapter sequences and PhiX with matching kmer length of 31 bp at a hamming distance of 1. Reads were merged using USEARCH (44) with a minimum length threshold of 175 bp and maximum error rate of 1%. Sequences were dereplicated (minimum sequence abundance of 2) and clustered using the distance-based, greedy clustering method of USEARCH (44) at 97% pairwise sequence identity among operational taxonomic unit (OTU) member sequences. *De novo* prediction of chimeric sequences was performed using USEARCH during clustering. Taxonomy was assigned to OTU sequences at a minimum identity cutoff fraction of 0.8 using the global alignment method implemented in USEARCH across RDP Trainset database version 15 trained with UTAX 250-bp configuration (45). OTU seed sequences were filtered against RDP Gold reference database version 9 to identify chimeric OTUs using USEARCH.

### Metagenome and metatranscriptome library preparation and sequence analysis.

DNA extracted from the technical replicates for each soil location prior to perturbations (nine samples in total) was pooled to construct a single long-hybrid Moleculo subassembly according to the manufacturer’s protocol (Illumina, San Diego, CA) and as previously described (46, 47). The data were assembled on BaseSpace using the Illumina TruSeq Long-Read Assembly application v1.0. Moleculo long hybrid read subassembly, annotation, mapping, and hybrid assembly have been previously described (47).

Metagenomic and metatranscriptomic libraries were constructed from technical replicates for treatment and control conditions, as applicable for the respective perturbation experiments. Libraries were prepared using 500 ng of DNA and 150 ng of cDNA. The DNA and cDNA were sheared to approximately 450 to 500 bp using a Covaris M220 focused-ultrasonicator instrument (Covaris M220 series; Woburn, MA). Sheared DNA fragments were end repaired, A tailed, and ligated to TruSeq adapters from Illumina according to the manufacturer’s instructions (Illumina, San Diego, CA). Adapter dimers were removed twice using Agencourt AMPure XP magnetic beads (Beckman Coulter, Danvers, MA). Libraries were checked for size and adapter dimers using a high-sensitivity DNA chip on a 2100 Bioanalyzer (Agilent Technologies, Santa Clara, CA) and quantified by qPCR (StepOne Plus system; Applied Biosystems, Foster City, CA) using the KAPA library quantification kit (KAPA Biosystems, Wilmington, MA) according to the manufacturer’s instructions. Fragments were sequenced on the HiSeq sequencing platform (Illumina, San Diego, CA) with 3 runs of 250-bp paired-end reads that generated ∼10 million reads per sample.

Replicate metatranscriptomes were quality controlled, screened for phiX, and mapped to the Moleculo subassembly using Bowtie 2 (48). Genes in the assembly were annotated with function and taxonomic rank using MetaPathways v.2.5 (49). Read counts per function for each condition were generated using Kyoto Encyclopedia of Genes and Genomes Orthology (KO) and Enzyme Commission (EC) numbers for both MTs and MGs using ATLAS (https://github.com/metagenome-atlas/atlas). The KO and EC count matrices were used for analyses of MG and MT functional data sets.

The aligned read abundances from the metatranscriptomes from the different treatments were used to determine which members of the community were active under the different conditions. The metatranscriptomes for each moisture condition from soils A and C were mapped to metagenomic contigs, containing 810,853 open reading frames, which were assembled from existing metagenome data from the same original soil batch (40). All metatranscriptomic reads in all samples were able to be aligned to the assembly. The average number of metatranscriptomic reads per sample that mapped to open reading frames on the assembly was 116,316.

### Metabolite extraction, analysis, and data processing.

We conducted an untargeted analysis of polar metabolites in soil using a direct extraction method (50). Briefly, ∼5 g wet soil was aliquoted into 50-ml screw-cap self-standing tubes (Next Advance, Averill Park, NY) containing 0.9- to 2.0-mm stainless steel beads, 0.1-mm zirconia beads, and 0.1-mm garnet beads and 8 ml of 60% (vol/vol) methanol in Nanopure water to effectively quench metabolic reactions upon lysis (51). Samples were bead beaten in a 50-ml Bullet Blender (Next Advance, Averill Park, NY) at speed 12 for 15 min at 4°C and subsequently transferred to chemical-compatible polypropylene 50-ml tubes (Olympus Plastics, Waltham, MA). Twelve milliliters of ice-cold chloroform was added, and each sample was probe sonicated at 60% amplitude (in a fume hood) for 30 s on ice, allowed to cool on ice, and sonicated again. The samples were allowed to completely cool at −80°C for ∼10 min and then centrifuged at 4,500 × *g* for 10 min at 4°C to separate the aqueous phase. The upper aqueous phases containing polar metabolites were transferred into glass vials using serological pipettes, dried down completely in a vacuum concentrator (Labconco, Kansas City, MO), and stored at −20°C until chemical derivatization. Details of chemical derivatization and gas chromatography-mass spectrometry (GC-MS) analyses have been previously published (52).

GC-MS raw data files were processed using MetaboliteDetector (53). Retention indices (RIs) of metabolites were calculated based on analysis of a mixture of fatty acid methyl ester (FAME) standards (C_8_ to C_28_), followed by their chromatographic alignment across all analyses after deconvolution. Metabolites were then identified by matching GC-MS features (characterized by measured RIs and mass spectra) to the Agilent Fiehn Metabolomics Retention Time Locked (RTL) library (54). All metabolite identifications were manually validated to reduce deconvolution errors during automated data processing and to eliminate false identifications. The relative abundance of metabolites was calculated based on the peak integration of three selected mass fragments from a single metabolite peak, and the information for three quantification ions was used consistently for all the samples in the study.

A total of 165 metabolites were measured by GC-MS from the 27 samples, including biological replicates from the three sites A, B, and C for the three treatments, wet, dry, and control. Of these, 70 could be identified using the reference databases. Evaluation for outlier behavior resulted in the removal of one replicate from soil C dry treatment. A one-factor analysis of variance (ANOVA) model with main effect of treatment was performed for each metabolite and site. *Post hoc* tests for three specific differences were run: wet versus dry, wet versus control, and dry versus control.

### Construction of metabolic network model.

We constructed the metabolic network model by assembling all reactions downloaded from the KEGG database (termed the master network). While the metabolic network *per se* is generic, the incorporation of context-specific (i.e., soil site- and moisture-dependent) multi-omics data led to the identification of sets of reactions that were differentially activated under the wet and dry conditions compared to the control. In comparison to stand-alone data analysis performed above, this integrative approach enabled illustration of the key biochemical changes based on an expanded set of reactions by determining the most probable paths that connect the measured metabolites and transcripts (see Fig. S5). Using this method, we identified 209 and 129 activated reactions derived from the 25 and 7 overexpressed transcripts under the dry and wet conditions, respectively, compared to the control conditions in soil A. For soil C, we identified 194 and 135 activated reactions using 13 and 5 genes overexpressed under dry and wet conditions, respectively. Soil B could not be part of this analysis due to the lack of metatranscriptomic data. Based on a hierarchy of reference pathways described in the KEGG database, we classified the identified reactions into reaction module levels (also see the supplemental material).

### Statistical analyses.

Estimates of *α*-diversity (observed, Chao 1, and inverse Simpson) based on 16S data were obtained from nonrarified data. *β*-Diversity analysis was based on upper 75th-quartile normalized library counts. Nonmetric multidimensional scaling or principal-coordinate analysis (PCoA) plotting was used to visualize *β*-diversity based on unweighted UniFrac or Bray-Curtis distances using functions supported by the package Phyloseq (55).

Count data generated from sequence analyses for MGs and MTs were screened for significant differences between treatments using a negative binomial frequency distribution model, as implemented in the package DESeq2 (56) and based on upper 75th-quartile normalized library counts. The most abundant transcripts were visualized as a heat map using a hierarchical clustering method implemented in the R package pheatmap. Differences in the abundance of taxa and the variance in expression of transcripts were characterized using the DESeq2 parameters fitType = “local” and an adjusted *P* value threshold of 0.05 to calculate log_2_ fold changes between treatments. The significant (*P* < 0.05) KOs from the MT data were subjected to pathway enrichment analyses using the package GAGE (57), and pathways were visualized using the package Pathview (58). Analysis was performed using the R programming language (v. 3.3.2) (59).

Abundance data for metabolites were log_2_(*x*) transformed and median centered. Data were processed on the log_2_ scale, and missing values were not imputed. Data were evaluated for outlier behavior using rMd-PAV (60), a robust principal-component analysis (PCA)-based approach, and any outliers were removed. A two-factor analysis of variance (ANOVA) model, with main effects of treatment and site, as well as an interaction of the two main effects, was performed for each metabolite to simultaneously evaluate the statistical hypothesis that there is equal metabolite abundance by treatment and site. The inclusion of an interaction term enabled a test of equal metabolite abundances for treatment within each site and the evaluation of different treatment effects (directions) across sites. Further, a PCA approach that allows missing values, projection pursuit PCA (61), was performed on metabolites that showed a significant treatment effect for at least one site. A total of 100 metabolites were used in computing the principal coordinates using methods described above after accounting for missing values using the abovementioned PPCA approach. Differences in metabolite relative abundances between the treatment groups were tested by Kruskal-Wallis test at the 0.05 level of significance. A *post hoc* pairwise comparison was performed using Bonferroni’s test. Additional description of the metabolite data analysis can be found in the supplemental material. A complete list of compounds identified in this study is provided in Table S3.

### Modeling approach for identifying active reaction modules under dry and wet conditions.

We identified distinct sets of reactions, i.e., reaction modules that were activated under the soil dry and wet conditions, by incorporating metabolite and transcript measurements into a metabolic network model. First, we constructed a metabolic network by collecting all reactions and metabolites obtained from the KEGG database (release date 28 May 2018). The resulting network included a total of 10,826 reactions and 8,398 compounds. We then converted reaction stoichiometry to binary representation so that the network model provides a complete description of connectivity between metabolites and reactions.

The network constructed as described above can be considered a generic model, which is applicable across treatment conditions. In order to identify the condition-specific (soil and moisture status combinations) activated pathways, we constrained the generic metabolic network to the metabolite and metatranscriptomic data collected from the control, dry, and wet conditions, respectively, for soils A and C. For measured metabolites, we forced the model to activate at least one of the associated reactions (involved in either their production or consumption). We further constrained the model by incorporating overexpressed genes under dry and wet conditions in comparison to control. For this purpose, we first identified the differentially expressed transcripts, i.e., EC numbers (dry versus control; wet versus control), using DESeq2 (56). We selected genes based on two criteria: (i) differential gene expressions that were statistically significant (i.e., false discovery rate [FDR] < 0.1) and (ii) expression levels under dry and wet conditions that were two times higher than those in control soils (i.e., log_2_ FC > 1). For multiple testing adjustment, the Benjamini-Hochberg (BH) (62) adjustment method was employed. For soil A, we identified 25 and 7 overexpressed genes for dry and wet conditions versus its control, resulting in 209 and 129 activated reactions, respectively. Similarly, we identified 13 and 5 genes overexpressed under dry and wet conditions for soil C, resulting in 194 and 135 activated reactions, respectively. We forced the model to activate the reactions associated with highly expressed genes thus identified.

Under the constraints described above, we formulated a Mixed Integer Linear Programming (MILP) problem to obtain minimal pathways or subnetworks that connect all metabolites and reactions incorporated (as constraints) into the model. We termed our algorithm “Metabolite-Expression-Metabolic Network Integration for Pathway Identification and Selection” (MEMPIS). We solved the formulated minimization problem using the IBM ILOG CPLEX version 12.7.1 optimization package.

For visualizing a set of predicted reactions and metabolites, after extracting the corresponding subnetwork from the generic model, the subnetwork was converted to an indirect bipartite graph with two types of nodes: reactions and compounds. We used Python package NetworkX and Gephi for graph manipulation and network visualization. Also, we used iPath3 (63) to visualize and compare the activated reactions between dry and wet conditions on KEGG global maps summarizing the comprehensive KEGG pathway maps and KEGG modules.

### Availability of data.

All data are made publicly available at the Open Source Framework (OSFHOME) and can be retrieved from https://osf.io/4uvj7/.
