# Supplementary figures and images for: Metaphenomic Responses of a Native Prairie Soil Microbiome to Moisture Perturbations
Source: mSystems. 2019 Jun 11;4(4):e00061-19. doi: 10.1128/mSystems.00061-19 (PMC6561317; doi:10.1128/mSystems.00061-19)

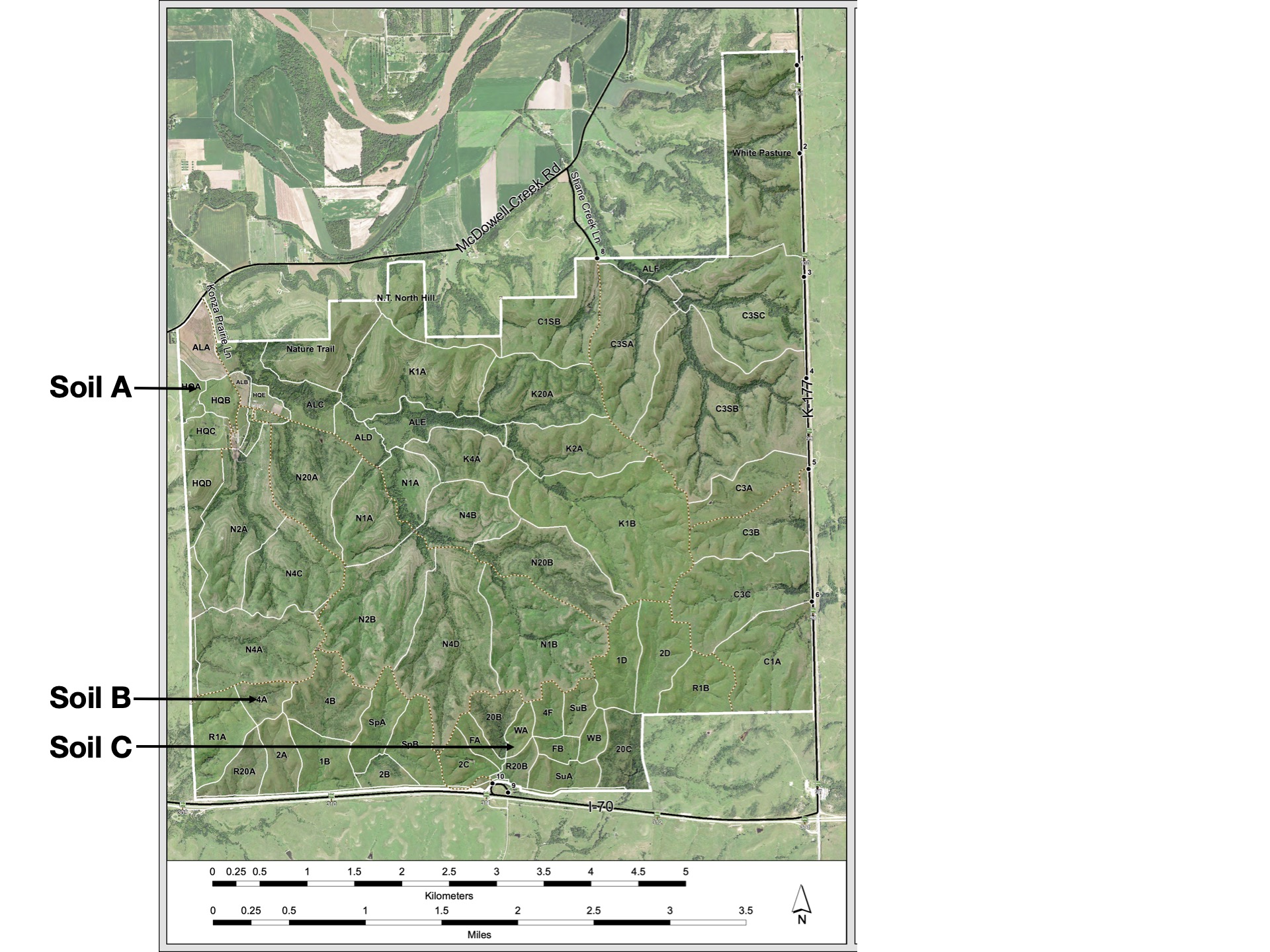

Supplement: FIG S1 [file mSystems.00061-19-sf001.jpg]

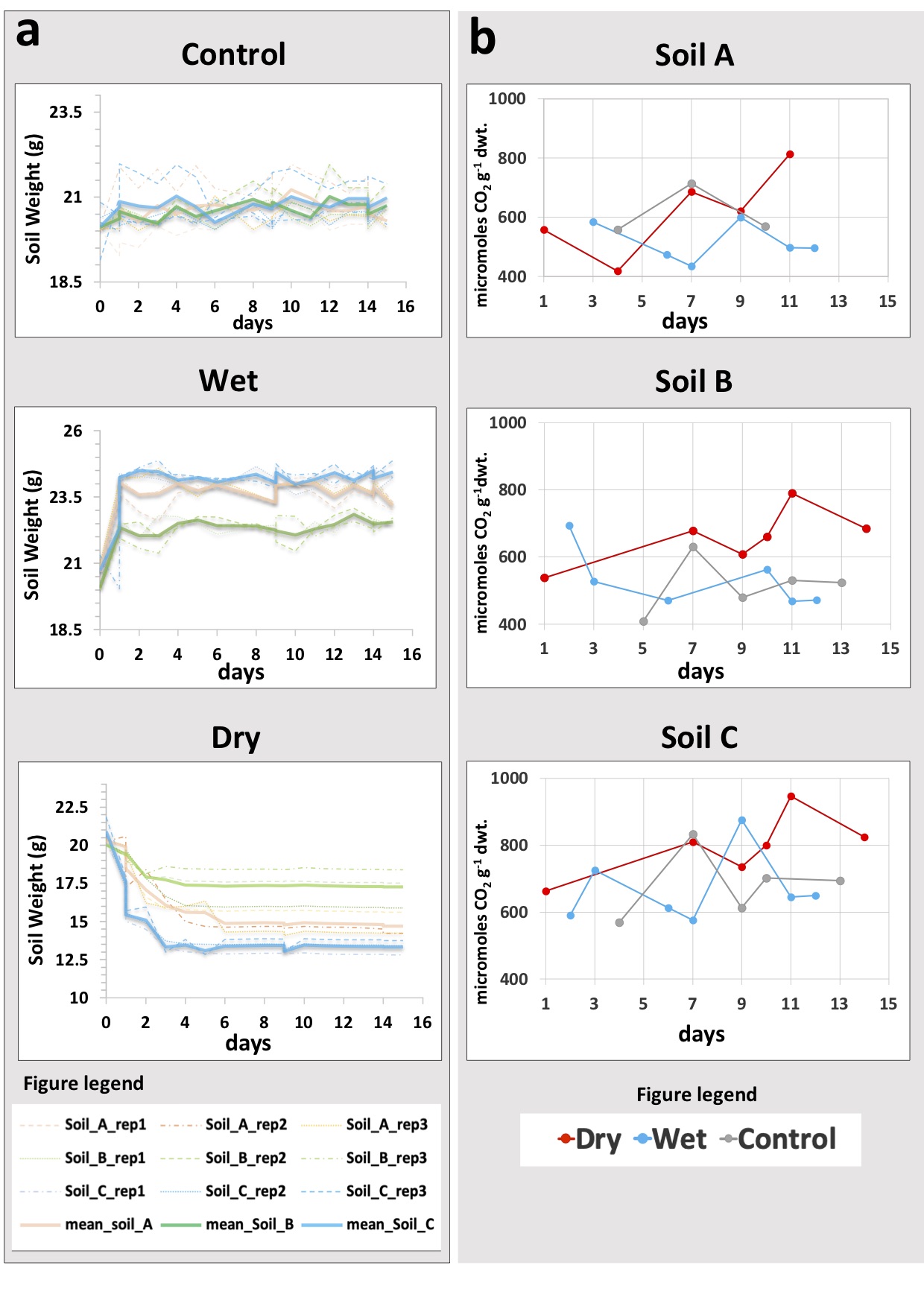

Supplement: FIG S2 [file mSystems.00061-19-sf002.jpg]

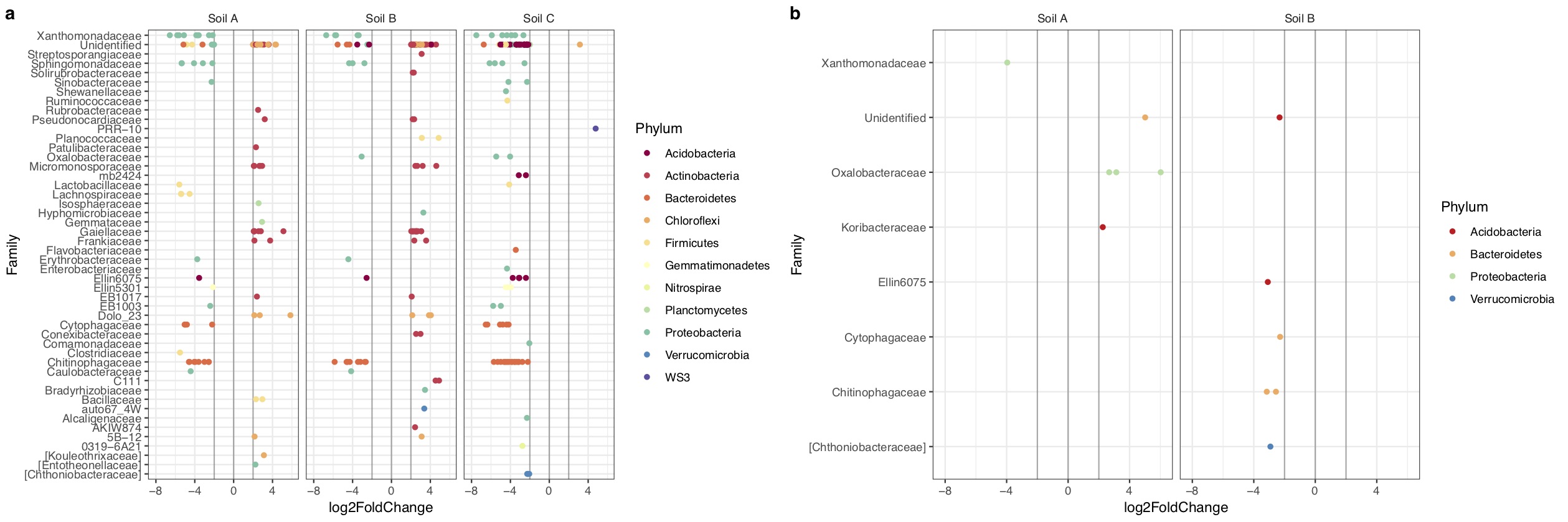

Supplement: FIG S3 [file mSystems.00061-19-sf003.jpg]

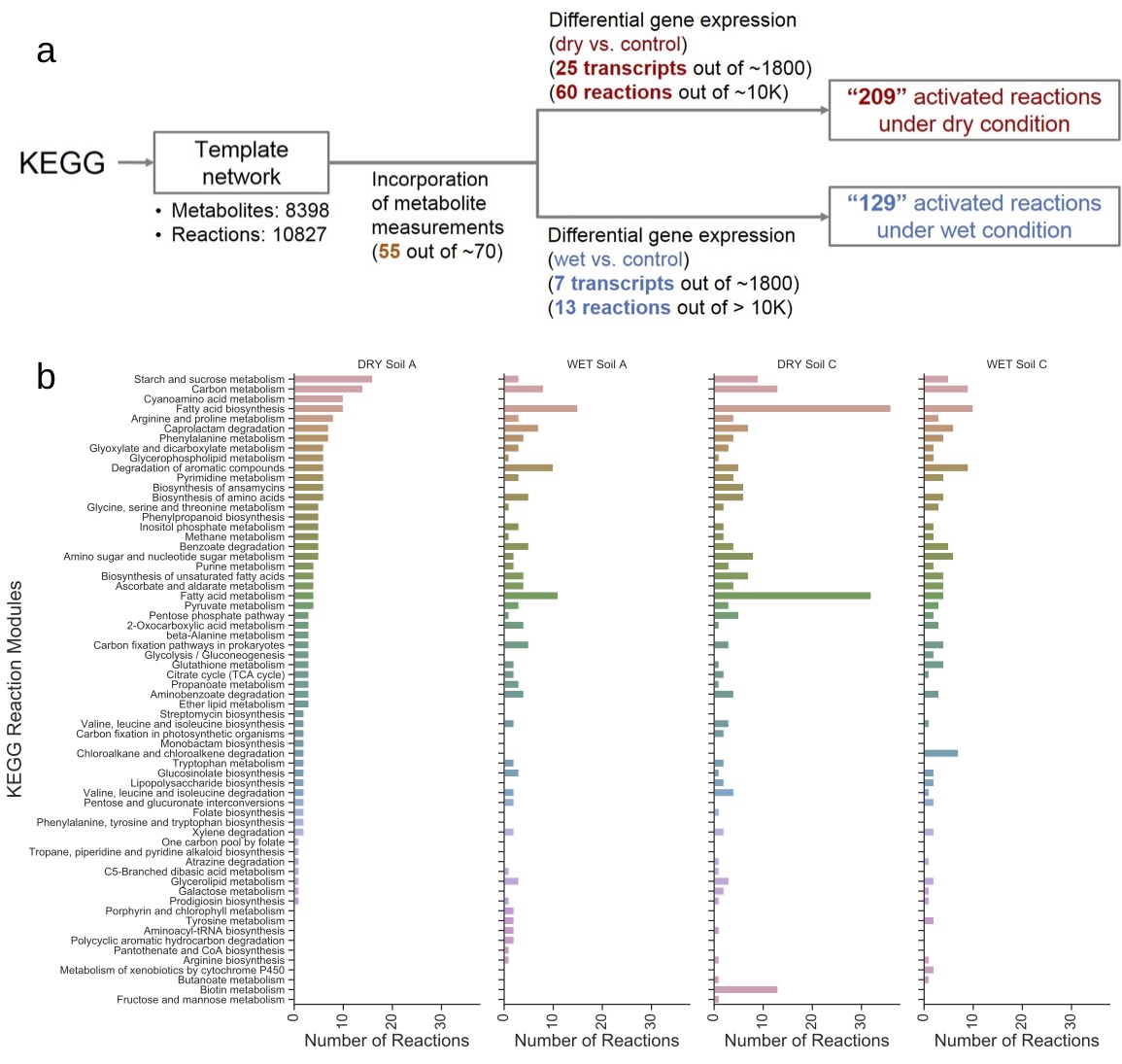

Supplement: FIG S5 [file mSystems.00061-19-sf005.jpg]
