# Supplementary material for: Metaphenomic Responses of a Native Prairie Soil Microbiome to Moisture Perturbations
Source: mSystems. 2019 Jun 11;4(4):e00061-19. doi: 10.1128/mSystems.00061-19 (PMC6561317; doi:10.1128/mSystems.00061-19)
Supplement: TABLE S1 [file mSystems.00061-19-st001.docx]

#### Table S1: Physico-chemical properties of soils collected from three locations in a watershed in the Kansas Native Prairie LTER station

| **Soil** | **pH (1:5_water_)** | **Moisture content** | **WFPS^*^ (Initial)** |  | **Clay** | **C:N** | **NO_3_-N^†^** | **SO_4_^2-^** | **WFPS-Wet^*^ (Final)** | **WFPS-Dry^*^ (Final)** |
| --- | --- | --- | --- | --- | --- | --- | --- | --- | --- | --- |
|  |  | **%** | **%** |  | **%** |  | **ppm** | **ppm** | **%** | **%** |
| A | 6.5 | 37 | 69.6 |  | 2 | 12 | 0.06 | 5.45 | 84.6 | 47 |
| B | 7 | 18 | 33.8 |  | 74 | 18 | 0.06 | 2.77 | 56.4 | 22.5 |
| C | 6.8 | 31 | 58.3 |  | 18 | 13 | 0.06 | 3.51 | 75.2 | 47 |

^*^Water-filled pore space (WFPS) estimated from gravimetric water content at the beginning (Initial) and end of incubation (Final) for Wet (WFPS-Wet) and Dry (WFPS-Dry) conditions; assumed soil bulk density (1.1 g cm^-3^) and particle density (g cm^-3^)

† Analytical detection limit for nitrate was 0.05 ppm
