# Supplementary material for: Metaphenomic Responses of a Native Prairie Soil Microbiome to Moisture Perturbations
Source: mSystems. 2019 Jun 11;4(4):e00061-19. doi: 10.1128/mSystems.00061-19 (PMC6561317; doi:10.1128/mSystems.00061-19)
Supplement: TABLE S2 [file mSystems.00061-19-st002.docx]

**Supplementary Table S2. Transcripts showing significant changes in response to drying relative to the wet conditions in soils A and C.**

Results are outputs from differential analysis of metatranscriptome count data annotated as Enzyme Commission (EC) numbers.

Columns show ECs (EC), The KEGG Enzyme Entry, the average of the normalized count values divided by size factors taken over all samples (baseMean), the effect size estimate (log_2_Fold Change), standard error estimate for the log_2_Fold Change estimates (lfcSE), *p*-values (pvalue), adjusted *p*-adjusted (padj). All values with an absolute value of log_2_Fold Change > 1.5, and *p-adj* < 0.05 are considered significantly changed in expression between the dry and wet treatments. Data were analyzed using the R package DESeq2.

| **Soil A** | | | | | | | |
| --- | --- | --- | --- | --- | --- | --- | --- |
| **EC** | **KEGG Enzyme Entry** | **baseMean** | **log2FoldChange** | **lfcSE** | **stat** | **pvalue** | **padj** |
| 1.2.2.2 | Oxidoreductases | 24.5854909 | -2.038857 | 0.38520391 | -5.2929292 | 1.20E-07 | 5.50E-05 |
| 1.2.1.46 | formaldehyde dehydrogenase | 19.3650718 | -1.9864078 | 0.41192562 | -4.8222487 | 1.42E-06 | 0.00018168 |
| 5.4.99.15 | **(1->4)-alpha-D-glucan 1-alpha-D-glucosylmutase** | 42.7241771 | -1.5378345 | 0.30272328 | -4.7496662 | 2.04E-06 | 0.01418168 |
| 5.4.99.16 | trehalose synthase | 97.7547563 | -1.5190846 | 0.27695017 | -4.3657116 | 1.27E-05 | 0.00062325 |
| 2.4.1.230 | kojibiose phosphorylase | 10.1804334 | -1.8660097 | 0.43150873 | -4.3243846 | 1.53E-05 | 0.00102291 |
| 6.4.1.1 | pyruvate carboxylase | 121.337137 | 1.85466293 | 0.43766363 | 4.23764461 | 2.26E-05 | 0.00109858 |
| 3.4.24.28 | Megateriopeptidase | 23.5864315 | -1.7168768 | 0.35636527 | -4.8177445 | 1.45E-06 | 0.00013046 |
| 3.1.4.4 | **Phospholipase D** | 12.1643717 | -1.5150067 | 0.4153462 | -3.6475756 | 0.00026473 | 0.00674422 |
| 3.4.15.5 | Dipeptidyl carboxypeptidase | 10.8284092 | 1.4825717 | 0.40961158 | 3.61945752 | 0.00029522 | 0.00709554 |
| 3.1.3.12 | Trehalose phosphatase | 12.1643717 | 1.5415937 | 0.42780178 | -3.6035233 | 0.00031393 | 0.03329437 |
|  |  |  |  |  |  |  |  |
|  |  |  |  |  |  |  |  |
|  |  |  |  |  |  |  |  |
|  |  |  |  |  |  |  |  |
|  |  |  |  |  |  |  |  |
| **Soil C** |  |  |  |  |  |  |  |
| **EC** | **KEGG Enzyme Entry** | **baseMean** | **log2FoldChange** | **lfcSE** | **stat** | **pvalue** | **padj** |
| 1.2.2.2 | Oxidoreductases | 24.5854909 | -2.3355001 | 0.39747049 | -5.875908 | 4.21E-09 | 3.31E-06 |
| 5.4.99.15 | **(1->4)-alpha-D-glucan 1-alpha-D-glucosylmutase** | 42.7241771 | -1.7542398 | 0.316 | -5.5513917 | 2.83E-08 | 1.12E-05 |
| 5.4.99.16 | trehalose synthase | 102.862211 | -1.589958 | 0.28519808 | -4.1723913 | 3.01E-05 | 0.00018496 |
| 3.4.21.108 | HtrA2 peptidase | 56.0638238 | -1.610456 | 0.29662729 | -5.4292239 | 5.66E-08 | 1.48E-05 |
| 3.4.24.28 | Megateriopeptidase | 22.728208 | -1.6511619 | 0.36671028 | -4.5026334 | 6.71E-06 | 0.00048019 |
| 1.2.1.60 | 5-carboxymethyl-2-hydroxymuconic-semialdehyde dehydrogenase | 201.05124 | 1.81296557 | 0.4499232 | 4.02950005 | 5.59E-05 | 0.00258764 |
| 1.1.1.274 | 2,5-didehydrogluconate reductase | 13.5096111 | -1.7645221 | 0.40132066 | -4.3967886 | 1.10E-05 | 0.00059846 |
| 1.2.1.51 | Pyruvate dehydrogenase | 13.2624523 | 1.54214209 | 0.41829746 | 3.68671161 | 0.00022717 | 0.00851349 |
| 3.1.4.4 | **Phospholipase D** | 12.1643717 | -1.5415937 | 0.42780178 | -3.6035233 | 0.00031393 | 0.01029437 |
| 1.1.1.40 | malate dehydrogenase | 8.64377059 | 1.55237017 | 0.44624532 | 3.47873713 | 0.00050378 | 0.01415989 |
| 3.1.3.12 | **Trehalose-phosphatase** | 16.4127976 | 1.7209713 | 0.35914611 | -2.0074596 | 0.00470075 | 0.046330426 |
