# Supplementary material for: Metaphenomic Responses of a Native Prairie Soil Microbiome to Moisture Perturbations
Source: mSystems. 2019 Jun 11;4(4):e00061-19. doi: 10.1128/mSystems.00061-19 (PMC6561317; doi:10.1128/mSystems.00061-19)
Supplement: FIG S4 [file mSystems.00061-19-sf004.docx]

**Supplementary Figure S4**: Box-plots showing relative abundances of (a) simple carbohydrates, (b) acids, (c) alcohols, and (d) amines, in response to the Dry or Wet treatments compared to Control conditions in soils A, B and C. Relative abundance measurements were obtained at the end of incubation of samples (t=15 days). Data were normalized by median-centering and log_2_ transformed. Whiskers indicate the most extreme values within 1.5 multiplied by the interquartile region. Box: 25 % quartile; median, 75 % quartile. Pairwise comparison of means to test treatment effects were performed after outlier removal.

|  |
| --- |
|  |
| Figure S4a. Simple carbohydrates |
| 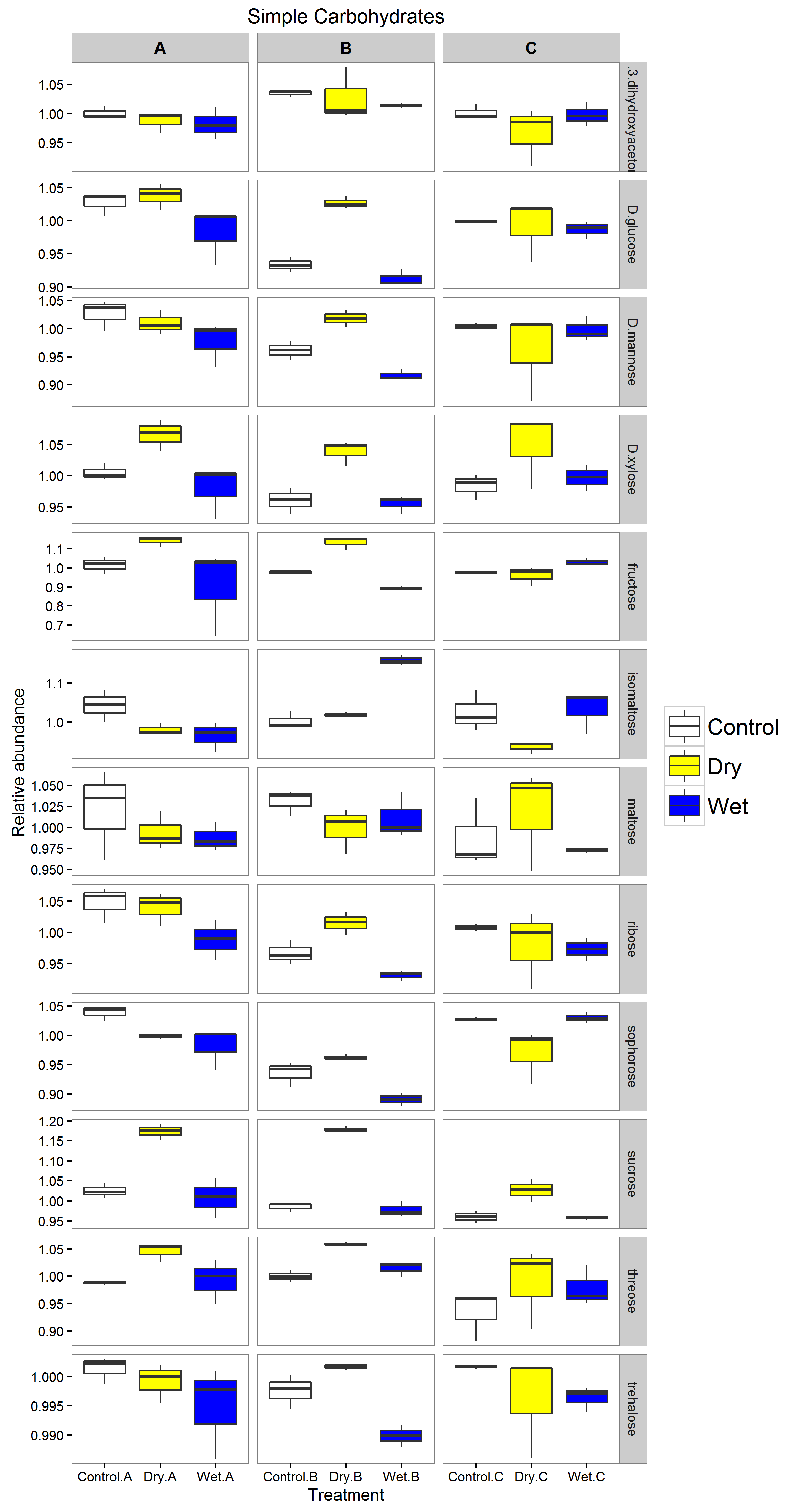 |
| Figure S4b. Acids |
| **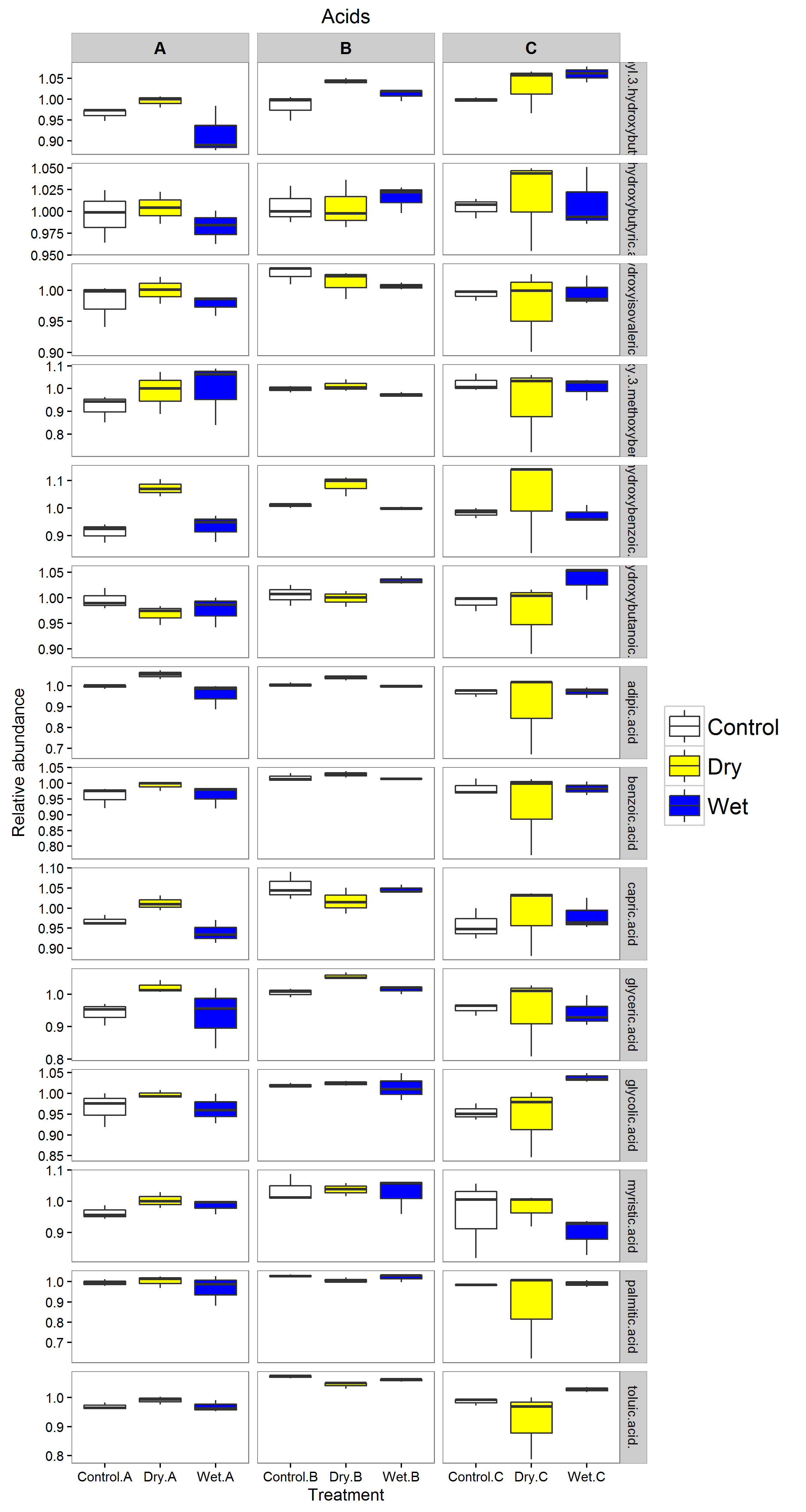** |
| Figure S4c. Alcohols |
| **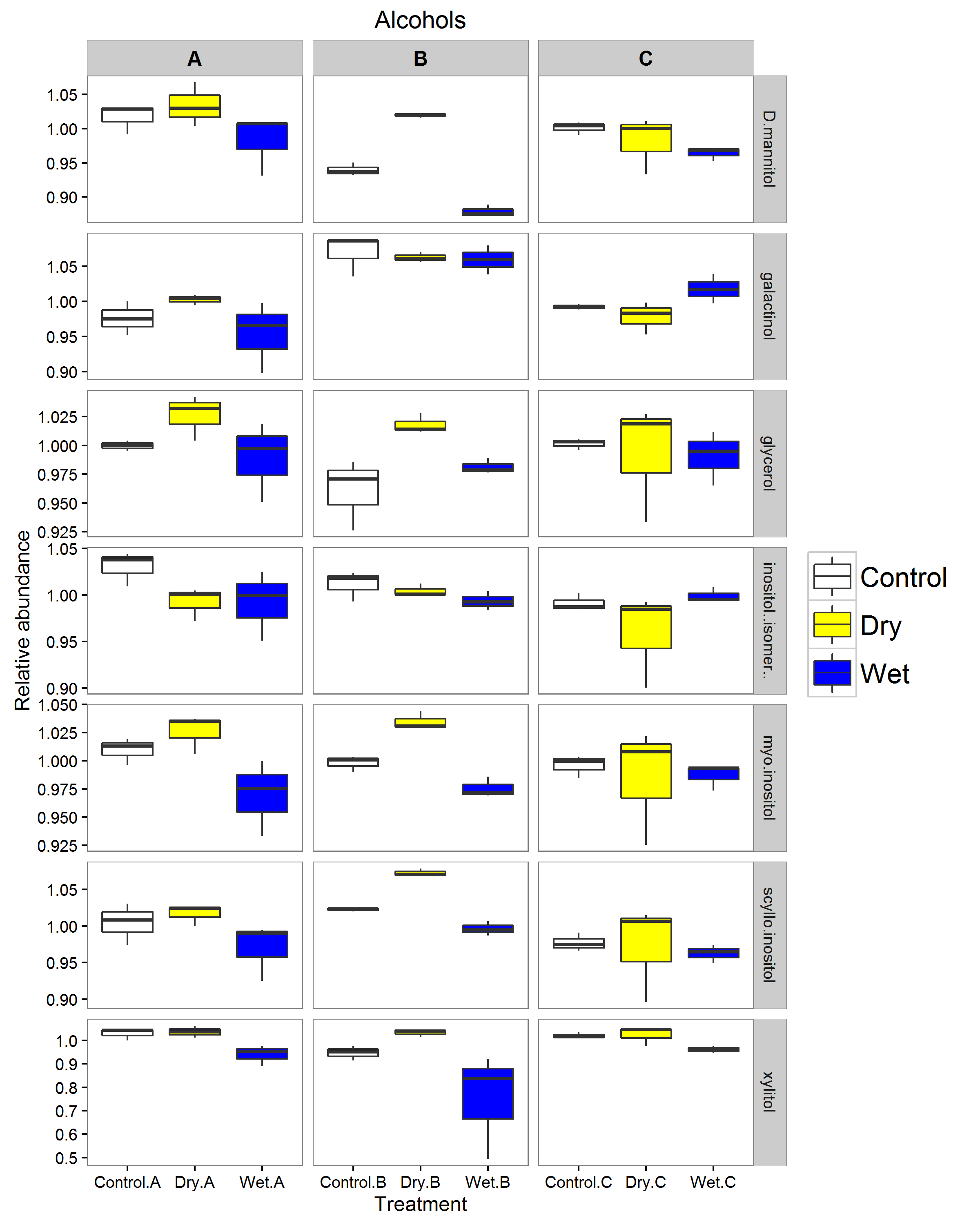** |
| Figure S4d. Amines |
| 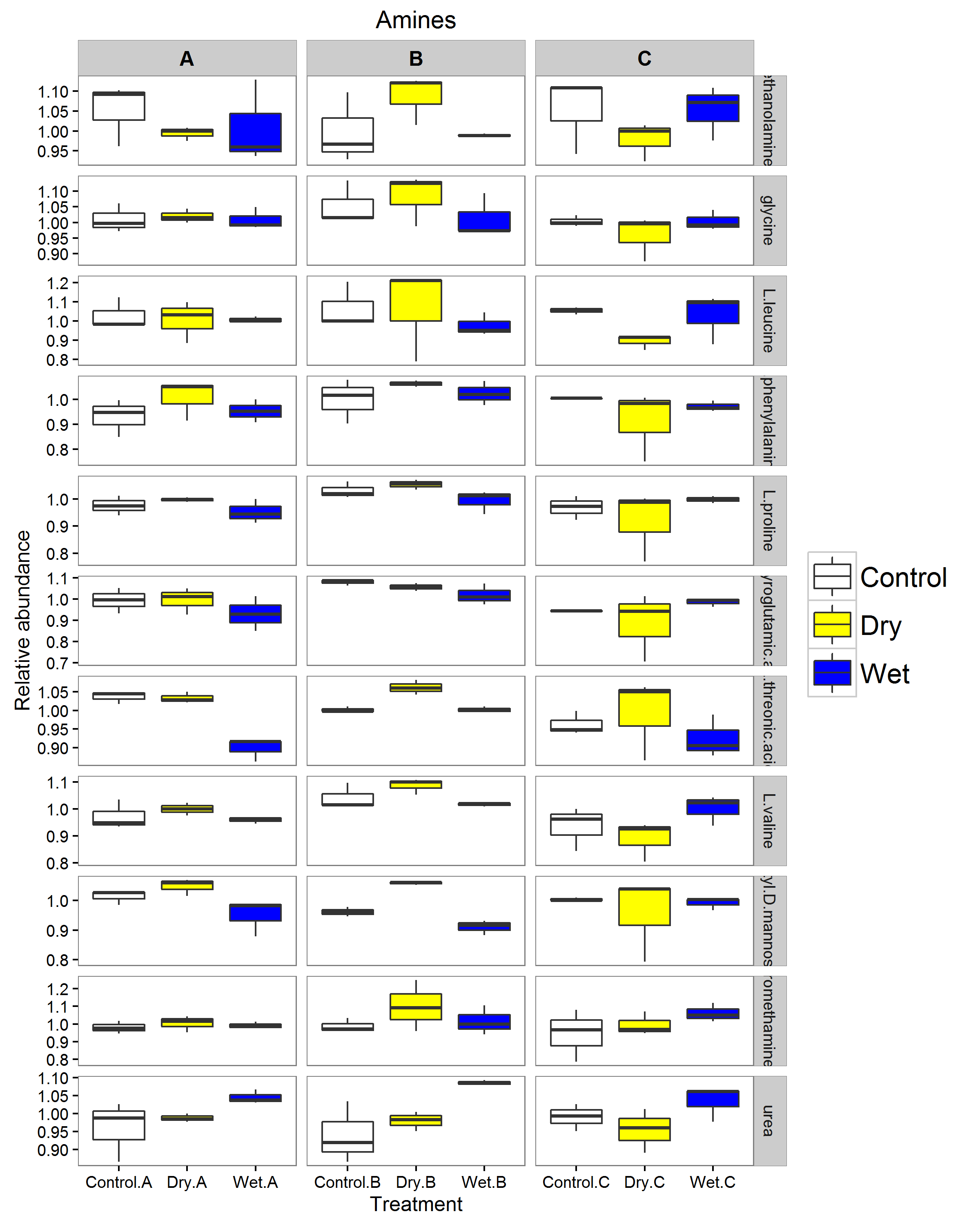 |
