# Supplementary material for: Metaphenomic Responses of a Native Prairie Soil Microbiome to Moisture Perturbations
Source: mSystems. 2019 Jun 11;4(4):e00061-19. doi: 10.1128/mSystems.00061-19 (PMC6561317; doi:10.1128/mSystems.00061-19)
Supplement: TABLE S3 [file mSystems.00061-19-st003.docx]

| **Table S3. List of metabolites that changed significantly in at least one soil in response to wetting and drying. Relative abundance data from GC-MS were log2 transformed and median centered, as reported here.** | | | | | | | | | | | | | | | | | | | | | | | | | | | | | | |
| --- | --- | --- | --- | --- | --- | --- | --- | --- | --- | --- | --- | --- | --- | --- | --- | --- | --- | --- | --- | --- | --- | --- | --- | --- | --- | --- | --- | --- | --- | --- |
| **Soil** | **Treatment** | **xylose** | | **glucose** | | **fructose** | | **isomaltose** | | **trehalose** | | **sucrose** | | **mannitol** | | **myo-inositol** | | **xylitol** | | **scyllo-Inositol** | | **NADM*** | | **threonic acid** | | **urea** | | **4HBA**** | | **toluic acid** |
| A | Control | 1.020 | | 1.038 | | 1.020 | | 1.083 | | 1.002 | | 1.045 | | 1.031 | | 1.013 | | 1.044 | | 1.008 | | 1.028 | | 1.045 | | 0.988 | | 0.940 | | 0.964 |
| A | Control | 1.000 | | 1.037 | | 1.059 | | 1.000 | | 1.003 | | 1.022 | | 1.029 | | 1.019 | | 1.045 | | 1.030 | | 1.027 | | 1.049 | | 0.866 | | 0.874 | | 0.960 |
| A | Control | 0.995 | | 1.007 | | 0.969 | | 1.046 | | 0.999 | | 1.007 | | 0.991 | | 0.997 | | 1.000 | | 0.974 | | 0.985 | | 1.017 | | 1.025 | | 0.924 | | 0.983 |
| B | Control | 0.962 | | 0.922 | | 0.967 | | 1.029 | | 0.994 | | 0.971 | | 0.932 | | 0.990 | | 0.951 | | 1.023 | | 0.946 | | 0.996 | | 0.866 | | 1.000 | | 1.067 |
| B | Control | 0.981 | | 0.946 | | 0.989 | | 0.990 | | 1.000 | | 0.992 | | 0.950 | | 1.004 | | 0.977 | | 1.025 | | 0.977 | | 1.010 | | 1.034 | | 1.013 | | 1.074 |
| B | Control | 0.939 | | 0.932 | | 0.979 | | 0.988 | | 0.998 | | 0.993 | | 0.936 | | 1.001 | | 0.915 | | 1.020 | | 0.960 | | 0.998 | | 0.920 | | 1.017 | | 1.077 |
| C | Control | 0.989 | | 1.000 | | 0.978 | | 1.081 | | 1.002 | | 0.943 | | 1.004 | | 1.004 | | 1.035 | | 0.991 | | 1.010 | | 0.999 | | 0.951 | | 0.986 | | 0.992 |
| C | Control | 0.961 | | 0.998 | | 0.970 | | 1.011 | | 1.001 | | 0.961 | | 0.991 | | 1.000 | | 1.015 | | 0.975 | | 0.999 | | 0.948 | | 1.026 | | 0.963 | | 0.995 |
| C | Control | 1.001 | | 0.999 | | 0.977 | | 0.979 | | 1.002 | | 0.974 | | 1.009 | | 0.984 | | 1.013 | | 0.966 | | 1.000 | | 0.941 | | 0.993 | | 1.001 | | 0.972 |
| A | Wet | 1.006 | | 1.006 | | 1.027 | | 0.996 | | 0.998 | | 1.010 | | 1.007 | | 0.976 | | 0.978 | | 0.990 | | 0.989 | | 0.917 | | 1.067 | | 0.972 | | 0.962 |
| A | Wet | 0.931 | | 0.933 | | 0.641 | | 0.923 | | 0.986 | | 0.957 | | 0.931 | | 0.933 | | 0.890 | | 0.925 | | 0.879 | | 0.863 | | 1.030 | | 0.878 | | 0.953 |
| A | Wet | 1.002 | | 1.007 | | 1.044 | | 0.973 | | 1.001 | | 1.057 | | 1.007 | | 1.000 | | 0.953 | | 0.995 | | 0.982 | | 0.917 | | 1.038 | | 0.949 | | 0.991 |
| B | Wet | 0.939 | | 0.906 | | 0.883 | | 1.147 | | 0.988 | | 0.962 | | 0.872 | | 0.972 | | 0.493 | | 0.987 | | 0.915 | | 1.010 | | 1.092 | | 1.005 | | 1.062 |
| B | Wet | 0.966 | | 0.905 | | 0.906 | | 1.156 | | 0.990 | | 0.972 | | 0.875 | | 0.969 | | 0.922 | | 0.995 | | 0.882 | | 1.000 | | 1.080 | | 0.998 | | 1.055 |
| B | Wet | 0.962 | | 0.928 | | 0.889 | | 1.173 | | 0.992 | | 1.000 | | 0.889 | | 0.986 | | 0.838 | | 1.006 | | 0.931 | | 0.996 | | 1.084 | | 0.997 | | 1.064 |
| C | Wet | 0.998 | | 0.990 | | 1.052 | | 1.067 | | 0.998 | | 0.962 | | 0.972 | | 0.993 | | 0.945 | | 0.964 | | 1.003 | | 0.906 | | 1.061 | | 0.956 | | 1.036 |
| C | Wet | 1.018 | | 0.998 | | 1.020 | | 1.064 | | 0.997 | | 0.953 | | 0.953 | | 0.994 | | 0.976 | | 0.949 | | 1.003 | | 0.988 | | 1.062 | | 1.011 | | 1.029 |
| C | Wet | 0.975 | | 0.972 | | 1.017 | | 0.968 | | 0.994 | | 0.959 | | 0.968 | | 0.974 | | 0.963 | | 0.974 | | 0.967 | | 0.879 | | 0.977 | | 0.959 | | 1.018 |
| A | Dry | 1.039 | | 1.017 | | 1.108 | | 0.968 | | 0.995 | | 1.153 | | 1.004 | | 1.006 | | 1.013 | | 1.000 | | 1.015 | | 1.022 | | 1.000 | | 1.043 | | 0.976 |
| A | Dry | 1.069 | | 1.041 | | 1.155 | | 0.974 | | 1.000 | | 1.191 | | 1.030 | | 1.035 | | 1.036 | | 1.024 | | 1.059 | | 1.050 | | 0.986 | | 1.069 | | 1.003 |
| A | Dry | 1.090 | | 1.055 | | 1.162 | | 0.997 | | 1.002 | | 1.176 | | 1.068 | | 1.037 | | 1.064 | | 1.025 | | 1.068 | | 1.028 | | 0.977 | | 1.104 | | 0.993 |
| B | Dry | 1.048 | | 1.019 | | 1.151 | | 1.017 | | 1.002 | | 1.173 | | 1.023 | | 1.044 | | 1.047 | | 1.079 | | 1.063 | | 1.081 | | 1.004 | | 1.099 | | 1.051 |
| B | Dry | 1.053 | | 1.024 | | 1.095 | | 1.025 | | 1.002 | | 1.176 | | 1.016 | | 1.031 | | 1.014 | | 1.070 | | 1.061 | | 1.059 | | 0.952 | | 1.111 | | 1.050 |
| B | Dry | 1.016 | | 1.038 | | 1.158 | | 1.014 | | 1.001 | | 1.187 | | 1.020 | | 1.029 | | 1.040 | | 1.068 | | 1.053 | | 1.042 | | 0.983 | | 1.043 | | 1.031 |
| C | Dry | 1.082 | | 1.018 | | 0.981 | | 0.943 | | 1.001 | | 1.054 | | 1.011 | | 1.022 | | 1.050 | | 1.006 | | 1.041 | | 1.062 | | 1.012 | | 1.140 | | 1.000 |
| C | Dry | 1.085 | | 1.021 | | 1.000 | | 0.946 | | 1.002 | | 1.027 | | 1.000 | | 1.008 | | 1.047 | | 1.015 | | 1.038 | | 1.050 | | 0.960 | | 1.141 | | 0.969 |
| C | Dry | 0.979 | | 0.938 | | 0.904 | | 0.919 | | 0.986 | | 0.997 | | 0.933 | | 0.925 | | 0.976 | | 0.896 | | 0.793 | | 0.866 | | 0.891 | | 0.837 | | 0.788 |
| Adjusted p- values from Bonferroni test of pairwise comparisons between Wet and Dry treatments versus the Control | | | | | | | | | | | | | | | | | | | | | | | | | | | | | | |
| B | Dry vs Con | 0.007 | | 0.000 | | 0.000 | | 0.759 | | 0.136 | | 0.000 | | 0.000 | | 0.005 | |  | | 0.000 | | 0.001 | | 0.004 | | 1.000 | | 0.017 | | 0.0013 |
| B | Wet vs Con | 1.000 | | 0.212 | | 0.010 | | 0.000 | | 0.011 | | 1.000 | | 0.000 | | 0.047 | |  | | 0.009 | | 0.034 | | 1.000 | | 0.042 | | 1.000 | | 0.4266 |
| B | Wet vs Dry | 0.005 | | 0.000 | | 0.000 | | 0.000 | | 0.001 | | 0.000 | | 0.000 | | 0.000 | |  | | 0.000 | | 0.000 | | 0.004 | | 0.142 | | 0.009 | | 0.0058 |
| A | Dry vs Con |  | |  | |  | | 0.132 | |  | | 0.004 | |  | |  | | 1.000 | |  | |  | | 1.000 | |  | | 0.007 | |  |
| A | Wet vs Con |  | |  | |  | | 1.000 | |  | | 1.000 | |  | |  | | 0.051 | |  | |  | | 0.001 | |  | | 1.000 | |  |
| A | Wet vs Dry |  | |  | |  | | 0.034 | |  | | 0.003 | |  | |  | | 0.036 | |  | |  | | 0.001 | |  | | 0.014 | |  |
| *N-acetyl-D-glucosamine **4-hydroxybenzoic acid | | |  | |  | |  | |  | |  | |  | |  | |  | |  | |  | |  | |  | |  | |  | |
